# A common polymorphism in the human immunoreceptor NKp65 determines ligand interaction, cell surface expression and function

**DOI:** 10.1371/journal.pone.0329454

**Published:** 2025-08-13

**Authors:** Julian Leonard Lino Heller, Yvonne Bartel, Catalin Schach, Angelina Kirsten, Felicitas Schlatter, Alexander Steinle

**Affiliations:** Institute for Molecular Medicine, Goethe-University Frankfurt, Frankfurt am Main, Germany; Sungkyunkwan University - Suwon Campus: Sungkyunkwan University - Natural Sciences Campus, KOREA, REPUBLIC OF

## Abstract

**Background:**

The human immunoreceptor NKp65 is specifically expressed by a subset of human innate lymphocytes, i.e., innate lymphoid cells group 3 (ILC3) and activates cellular cytotoxicity upon interaction with its genetically linked ligand KACL. In the context of the present study, the relevance of a common polymorphism in the NKp65-coding *KLRF2* gene was addressed.

**Methods:**

Using biophysical methods such as flow cytometry and surface resonance spectroscopy, as well as immunological methods such as ELISA and immunoblots and cytotoxicity assays, the influence of polymorphism rs576601 on surface expression, binding kinetics to the ligand KACL, proteolytic cleavage, intracellular retention, and functional responsiveness of NKp65 was investigated.

**Results:**

Polymorphism rs576601 entails the exchange of cytosine for adenine, resulting in a substitution of proline by threonine at amino acid position 131 within the C-type lectin-like ectodomain of NKp65. Here, we show that the NKp65-Thr_131_ variant is only weakly expressed on the cell surface as compared to the NKp65-Pro_131_ variant. This is due to an enhanced intracellular retention of NKp65-Thr_131_ but not due to proteolytic cleavage. In addition, the polymorphism rs576601 has a significant impact on the binding kinetics and affinity to the ligand KACL. On a functional level, the combination of reduced surface expression and affinity resulted in a drastically reduced cellular cytotoxicity of NKp65-Thr_131_^+^ effector cells towards KACL^+^ target cells.

**Conclusions:**

The polymorphism rs576601 affects ligand interaction, cell surface expression and function of NKp65. Since the NKp65 ligand KACL is primarily expressed on keratinocytes, polymorphism rs576601 may impact on skin immunosurveillance by NKp65-expressing ILC3.

## Introduction

We previously characterized NKp65 and keratinocyte-associated C-type lectin (KACL) as an immune-related C-type lectin-like receptor (CTLR)-ligand system [1] encoded by the genes Killer Cell Lectin-Like Receptor Subfamily F Member 2 (*KLRF2,* NKp65) and C-Type Lectin Domain Family 2 Member A (*CLEC2A*, KACL) in a tail-to-tail orientation in the Natural Killer Gene Complex (NKC) on human chromosome 12 [2]. KACL transcripts were almost exclusively detected in human skin but also in some myeloid cell lines [3]. Accordingly, KACL surface expression was demonstrated for keratinocytes as well as for myeloid cell lines U937 and AML01 [1]. In contrast, expression of NKp65 is limited to the natural killer (NK) lymphoma cell line NK92-MI [1,4] as well as innate lymphoid cells group 3 (ILC3) [5]. ILC3 are situated in several organs including skin [6]. Hence, the NKp65-KACL axis may mediate immunosurveillance of human skin [7] by ILC3.

Upon engagement of cell-bound KACL, NKp65 stimulates release of proinflammatory cytokines and cellular cytotoxicity [1,5]. Activating NKp65 signaling depends on tyrosine 7 – embedded within a cytoplasmic hemi-immunoreceptor tyrosine-based activation motif (hemITAM) – and spleen tyrosine kinase (Syk) [1,8].

Mariuzza and colleagues [9] reported the crystal structure of the KACL-NKp65 complex. It showed that the combination of high shape complementarity (S_C_ of 0.69), high abundance of intermolecular hydrogen bonds [9] and hotspot amino acid residues [10] results in exceptionally high affinity of the KACL-NKp65 complex in the low nanomolar range [1,9].

Two prevalent coding polymorphisms, namely rs576601 (encoding for threonine versus proline at amino acid position 131) and rs1797517 (encoding for valine versus isoleucine at position 68) are localized within the NKp65 extracellular C-type lectin-like domain (CTLD) and the NKp65 stalk region, respectively (*https://www.ncbi.nlm.nih.gov/snp/*). According to the KACL-NKp65 complex structure, rs576601 is situated in the NKp65-KACL interface [9] and thus likely affects receptor-ligand interaction. On the other hand, rs1797517 localizes to an N-linked glycosylation motif in the NKp65 stalk region and therefore may effect protein glycosylation.

In the current study, we investigated the relevance of these NKp65 polymorphisms on NKp65-KACL interaction, NKp65 surface expression and functional recognition of KACL by NKp65.

## Materials and methods

### Recombinant proteins and antibodies

Initially, NKp65 cDNA was isolated from human NK cells [1]. Alterations in the NKp65 glycoprotein were introduced using site-directed mutagenesis of the NKp65 cDNA. Soluble recombinant NKp65 (Ser_63_ through Val_207_) was produced upon transient transfection in 293F cells using a pSecTag2 vector (Thermo Fisher Scientific, Waltham, Massachusetts, USA) containing the NKp65 cDNA, a C-terminal hexa-histidine and a c-myc tag as well as a N-terminal BirA tag [11,12]. The soluble rNKp65 ectodomains were isolated from culture supernatant using HisPur^TM^ nickel-nitrilotriacetic acid (Ni-NTA) agarose columns (Thermo Fisher Scientific) by gravity-flow and biotinylated enzymatically via BirA ligase (BioCat GmbH, Heidelberg, Germany). NKp65-specific monoclonal antibody (mAb) OMAR1 was described previously [5]. OMAR2 was generated by standard hybridoma technology. In brief, BALB/c mice were immunized with both sNKp65 and P815-NKp65, and resulting hybridoma screened for specific reactivity against 293 cell expressing an NKp65-pIRES-eGFP construct. Mice were housed at the animal facility of the faculty of Medicine of the Goethe University at 22 ± 2 °C with a 12:12 hour light-dark cycle in ventilated cages with an automatic watering system. Mice were sacrificed by cervical dislocation to obtain spleens. Animal experiments were reviewed and approved by the Regierungspräsidium Darmstadt (F146/Anz.03).

### Degranulation assay

NK92-MI effector cells were labeled using CellTrace™ FarRed (Thermo Fisher Scientific) according to manufacturer’s instructions. In brief, cells were washed thoroughly in phosphate-buffered saline (PBS) and adjusted to 1 x 10^6^ cells/ml. CellTrace™ FarRed was added (1:1000 dilution) followed by 20 min incubation at 37 °C protected from light. Labeling was stopped by adding complete media containing Bovine Calf Serum (BCS) and centrifugation to get rid of excessive dye. The assay was set up using 1 x 10^5^ effector cells and 0.33 x 10^5^ KACL-expressing U937 target cells (3:1) in a total volume of 200 µl in a U-bottom plate. Phycoerythrin (PE) conjugated anti-human CD107a antibody (Becton Dickinson (BD), Franklin Lakes, New Jersey, USA) was added (20 µl per well) followed by 1 h incubation at 37 °C in a humidified incubator. GolgiStop™ was added to reach a concentration of 1 µl per 1.5 ml total reaction volume. Therefore, a 10X stock was prepared (6.66 µl/ml) and 20 µl were added to each well. The assay was incubated for three more hours. Finally, cells were washed and resuspended in a final volume of 100 µl FACS buffer containing DAPI (100 ng/ml). CD107a expression of viable effector cells was analyzed by flow cytometry.

### GranToxiLux^®^ granzyme B delivery assay

Granzyme B delivery of NK92-MI towards target cells was quantified using the GranToxiLux® Assay Kit (OncoImmunin, Gaithersburg, Maryland, USA) [13]. U937 target cells were fluorescently labeled according to manufacturer’s instructions. In brief, 1 x 10^6^ cells were washed and incubated for 20 min at 37 °C in 500 µl Medium T containing Target Fluorescent Label-4 (TFL-4, dilution 1:1000). Excessive dye was removed by repeated washing in complete media. NK92-MI effector cells were mixed with labeled U937 target cells in 75 µl GranToxiLux^®^ substrate in a U-bottom plate at an E:T ratio of 16:1, reaching 3.2 x 10^5^ to 0.2 x 10^5^ cells per well, followed by 4 h incubation at 37 °C. For analysis, cells were washed using Wash Buffer before analysis by flow cytometry.

### CytoTox 96^®^ non-radioactive cytotoxicity assay

The Cytotoxicity Assay Kit (Promega, Madison, Wisconsin, USA) was used to determine the amount of lysed cells, which is proportional to the release of lactate dehydrogenase (LDH). The co-culture was set up using E:T ratios of 8:1–2:1 thereby reaching a cell count of 1.6 to 0.4 x 10^5^ effector and 0.2 x 10^5^ CEM.NKR target cells, respectively, in a total volume of 200 µl complete media, followed by 4 h incubation at 37 °C in a humidified incubator. To measure the LDH release, 50 µl of co-culture supernatant were incubated with 50 µl of CytoTox 96^®^ reagent for 30 min at room temperature (RT) protected from light. Finally, by adding 50 µl of Stop Solution, the reaction was stopped before measuring the absorption at 450 nm. Percent cytotoxicity was calculated according to manufacturer’s instruction: spontaneous LDH release of both, effector and target cells, was subtracted from experimental values and, subsequently, set in relation to the maximum LDH release of the target cells.

### Surface plasmon resonance (SPR)

Using a Biacore^TM^ X100 apparatus, varying response units (RU) of biotinylated rNKp65 molecules (22.3–89.5) were immobilized to the surface of Sensor Chip SA (Cytiva, Marlborough, Massachusetts, USA). To measure affinity and kinetics, immobilized NKp65 (imNKp65) was pulsed (flow rate, 30 µl/min) with soluble KACL ectodomains (0.247–150 nM) or mAbs OMAR1 and OMAR2 (1.25–20 nM), respectively. RU from FC2 flow chamber (reference chamber), containing only running buffer and imNKp65, were subtracted to calculate KACL-specific RU from the main flow cell. The response units of the analytes are illustrated as black traces whereas colored traces represent the fitting of a bivalent model. Raw data were analyzed and visualized using Biacore X100 Evaluation Software (Version 2.0.2 Plus Package).

### DAPI-based cytotoxicity assay

Cellular cytotoxicity of NK92-MI effector cells towards KACL-expressing U937 target cells was quantified using 4’,6-Diamidino-2-phenylindol (DAPI), a nucleic acid stain which is essentially excluded from viable cells. To distinguish NK92-MI effector and U937 target cells, the latter were marked using CellTrace™ FarRed (Thermo Fisher Scientific) according to manufacturer’s instructions. In brief, cells were washed thoroughly in PBS and adjusted to 1 x 10^6^ cells/ml. CellTrace™ FarRed was added (1:1000 dilution) followed by 20 min incubation at 37 °C protected from light. Labeling was stopped by adding complete media containing BCS and centrifugation to get rid of excessive dye. The assay was set up using different E:T ratios ranging from 4:1–16:1. Therefor, 0.4 x 10^5^ to 1.6 x 10^5^ NK92-MI cells were cultured with 0.1 x 10^5^ U937 cells at a volume of 100 µl in a U-bottom plate for 4 h in a humidified incubator. To demonstrate NKp65-specific cytotoxicity, the anti-NKp65 mAb OMAR1 (or an IgG1 isotype control of irrelevant specificity) was added at a concentration of 10 µg/ml to specifically block NKp65-KACL interaction. Finally, DAPI was added at a concentration of 33.3 ng/ml immediately before flow cytometric measurement of the sample. By gating on FarRed^+^/DAPI^+^ cells, the count of lysed target cells was determined. To calculate percent cytotoxicity, the counts were set in relation to control wells containing only U937 cells.

### Transfection

Approximately 24 h before transfection, FreeStyle™ 293-F cells were cultured at a density of 0.7 x 10^6^ cells/ml in FreeStyle^TM^ 293 expression media (Thermo Fisher Scientific). The flasks were placed on an orbital shaker rotating at 120 rpm in a humidified incubator at 37 °C with 8% CO_2_. On the day of transfection, cells were adjusted to 1 x 10^6^ cells/ml ensuring that cell viability exceeded 90%. Depending on the experiment, either 2 ml (NKp65 test expression) or 250 ml (rNKp65 production) were seeded into a 6-well plate or a 1 l Schott flask, respectively. Vector DNA and polyethylenimine (PEI) transfection reagent (Sigma-Aldrich, St. Louis, Missouri, USA) were equilibrated at RT. Per 1 x10^6^ cells, 2 µg DNA and 6 µg PEI (1:3) were diluted in 100 µl of supplement-free culture medium, respectively. Diluted DNA was added to PEI and after 20 min incubation at RT, the DNA-PEI mixture was added dropwise to the 293-F cells followed by 72 h incubation on an orbital shaker rotating at 120 rpm in a humidified incubator at 37 °C with 8% CO_2_.

### Transduction

On day one, 5 x 10^6^ Phoenix-AMPHO cells were seeded in a 10 cm cell culture dish in 10 ml complete Dulbecco’s Modified Eagle Medium (DMEM, Thermo Fisher Scientific) and cultured in a humidified incubator at 37 °C and 5% CO_2_ overnight. The next day, transfection was carried out as described above for 293-F cells, using 7 x 10^6^ cells per dish, followed by overnight incubation. One day prior to transduction of NK92-MI target cells (day 3), media was substituted by 5 ml Iscove’s Modified Dulbecco’s Medium (IMDM, Thermo Fisher Scientific). Transduction of NK92-MI target cells was performed on two consecutive days: Virus particles were isolated from supernatant by filtration (0.2 µm) and mixed with polybrene (Sigma-Aldrich) to achieve a final concentration of 8 µg per ml (day 4). NK92-MI cells were adjusted to 1 x 10^6^ cells/ml. For each condition, 100 µl of effector cells were distributed in duplicates into two wells of a 24-well plate and mixed with 500 µl of supernatant containing the virus particles. The mixture was centrifuged for 1.5 h at 900 x *g* at 32 °C, followed by a 2 h incubation in a humidified incubator at 37 °C and 5% CO_2_. Then, 0.5 ml IMDM medium was added and cells were incubated overnight. The next day (day 5), replicates were pooled and centrifuged for 3 min at 500 x *g.* Supernatant was discarded and the pellet was reconstituted in 2 ml of fresh supernatant from day 4 containing virus particles. The mixture was distributed onto 4 wells of a 24-well plate and centrifuged for 1.5 h at 900 x *g* at 32 °C, followed by 2 h incubation in a humidified incubator at 37 °C and 5% CO_2_. Then, 0.5 ml IMDM medium was added and cells were incubated for 2–3 days. Finally, cells were tested for NKp65 expression by flow cytometry and selection antibiotics were gradually added.

### ELISA

Soluble NKp65 (sNKp65) from 293-F cell culture supernatant was quantified using a sandwich Enzyme-linked Immunosorbent Assay (ELISA). A High Binding ELISA plate (Sarstedt, Nümbrecht, Germany) was coated overnight at 4 °C with 100 µl PBS per well containing 1 µg/ml anti-NKp65 capture antibody OMAR2. Unspecific binding sites were blocked for 1 h at 37 °C by adding 100 µl of 15% bovine serum albumin (BSA) per well. The mixture of antibody and BSA was discarded, followed by four consecutive washing steps using 200 µl PBS with Tween® (PBST). Subsequently, samples were loaded onto the plate and incubated for 2 h. Afterwards, samples were discarded, followed by four consecutive washing steps using 200 µl PBST. 100 µl of biotinylated anti-NKp65 sandwich antibody OMAR1 were added for 2 h at a concentration of 1 µg/ml, allowing binding to captured NKp65 at a different epitope than OMAR2. The sandwich antibody was discarded and the wells were washed by four consecutive washing steps. Streptavidin-conjugated horseradish peroxidase (SA-HRP, Jackson ImmunoResearch, Philadelphia, Pennsylvania) was diluted in PBS according to manufacturer’s instruction (1:10,000). 100 µl of the dilution were added to each well and incubated for 1 h. The plate was washed thoroughly by six consecutive washing steps, using 200 µl PBST. Finally, the colorimetric assay was completed by adding 100 µl of 3,3′,5,5′-Tetramethylbenzidine (TMB) 2-Component Peroxidase Substrate (LGC SeraCare, Milford, Massachusetts, USA) per well. Substrate turnover by HRP was terminated by adding 1 M phosphoric acid (Merck, Darmstadt, Germany) and absorbance at 450 nm was measured on a Thermo Scientific MULTISKAN FC device. Data was analyzed using the SkanIt Software (Version 2.5.1.4).

To quantify human IFN-γ and TNFα in the supernatant of co-cultures (E:T ratio 8:1), a respective ELISA Antibody Pair Set (Sino Biological, Beijing, China) was used according to the manufacturer’s instructions. For this purpose, 2.5 x 10^6^ effector cells and 0.3 x 10^6^ target cells were incubated in 1 ml RPMI1640 supplemented with 1% BCS for 4 h at 37 °C. The capture antibody was coated onto a high binding ELISA plate (Sarstedt, Nümbrecht, Germany) at a concentration of 2 µg/ml in 100 µL PBS and incubated overnight at 4 °C. Each well was washed three times with 200 µL PBST and blocked with 200 µl of 2% BSA in PBS for 1 h at RT. After three additional washing steps with PBST, 100 µl of samples or standards diluted in 0,1% BSA/PBS were added and incubated for 2 h at RT. Following three washing steps with PBST, 100 µl of the detection antibody in 0.1% BSA/PBS was added per well and incubated for 1 h at RT. After another three consecutive washing steps with PBST, the TMB substrate solution was added and the colorimetric detection was performed as described for the NKp65 ELISA. Wells containing single cultures of either effector or target cells served as control to subtract spontaneous release from co-culture.

### Immunoblotting

293-F transfectants were lysed with 100 µl NP40 buffer per 10^6^ cells. Protein content was determined by Bradford protein assay. 20 µg of protein was denatured for 10 min at 95 °C and deglycosylated by a 1 h treatment with 50 units of endoglycosidase (Endo) Hf (New England Biolabs Inc., Ipswich, Massachusetts, USA) at 37 °C. Samples were separated under reducing conditions in 15% sodium dodecyl-sulfate polyacrylamide gel electrophoresis (SDS-PAGE). Protein was transferred from the gel onto a 0.2 µm polyvinylidene fluoride (PVDF) membrane (Carl Roth, Karlsruhe, Deutschland) for 2 h at 0.8 mA/cm^2^. Unspecific binding sites were blocked with 5% milk powder in Tris-buffered saline with Tween® (TBST) overnight at 4°C. Anti-FLAG (M2) tag mAb [5 µg/ml] in 5 ml TBST was added for 2 h at RT. Secondary goat-anti-mouse Ig antibodies conjugated with HRP (Jackson ImmunoResearch, Philadelphia, Pennsylvania, USA) in 15 ml TBST (1:10,000) was added for 2 h at RT and binding was visualized using 1 ml HRP Juice PLUS and a Peqlab FUSION SL apparatus. Membrane was stripped in 5 ml Re-Blot Plus Mild (Merck, Darmstadt, Deutschland) for 15 min and blocked overnight as described. Anti-beta-actin antibodies (Sigma-Aldrich) conjugated with HRP diluted in TBST (1:10,000) were used for 2 h to detect actin. Data were visualized as described.

### Flow cytometry

NKp65 expression of 293-F transfectants and NK92 MI transductants was detected using a FACSCanto II cytometer (Becton, Dickinson and Company, Franklin Lakes, New Jersey, USA) and analyzed using FlowJo V10 software. Cells were transferred to 96-well plates or U-bottom tubes and centrifuged at 500 x *g* for 3 min, followed by resuspension in PBS and viability staining using the fixable viability dye (FVD) eFluor™ 506 (Thermo Fisher Scientific) according to manufacturer’s instructions. In brief, cells were kept at a concentration of 1–10 x 10^6^/ml. 1 µl of FVD was added per 1 ml of cells and vortexed immediately, followed by 30 min incubation at 2–8 °C, protected from light. Afterwards, cells were washed twice in FACS buffer and split in equal parts for intracellular and extracellular stainings. Cells for intracellular analyses were fixed with Cytofix/Cytoperm solution (BD Biosciences, Franklin Lakes, New Jersey) for permeabilization of the membrane for 20 min (4 °C, in the dark). From this step onward, samples for intracellular analyses were washed using Saponin buffer (Roth, Karlsruhe, Germany) while samples for extracellular analyses were washed with FACS buffer. Both types of samples were stained with primary antibodies OMAR1 and OMAR2 (10 µg/ml in either FACS or Saponin buffer) as well as anti-FLAG tag mAb M2 (10 µg/ml in either FACS or Saponin buffer) (Sigma-Aldrich) for 20 min, washed two times, followed by incubation with 1:100 diluted secondary APC-conjugated goat anti-mouse IgG (Jackson ImmunoResearch) on ice for again 20 min, protected from light.

### Gene targeting by CRISPR/Cas9

A NK92-MI *KLRF2* (NKp65) knockout cell line was generated according to Cao et al. [14]. In brief, 3 sgRNA against *KLRF2* (exon 2, 3 and 6, respectively) were designed using CHOPCHOP software [15] and incorporated during primer set design. Guide RNA was introduced into multi-guide-vector using golden-gate assembly. 293T Phoenix Ampho cells were transfected with lenti-iCas9-neo vector to produce virus particles. NK92-MI cells were transduced and sorted for the GFP+ population upon 24 h pre-treatment with doxycycline.

### Statistical analysis

Statistical analyses were conducted using the program “GraphPad Prism” Version 7.03 for Windows (Boston, Massachusetts, USA). Detailed descriptions are indicated in the corresponding figure legends. P-values are indicated by numbers, except p-values lower than 0.001 (***) and 0.0001 (****), according to statistical reporting guidelines.

## Results

### A common polymorphism in the NKp65 ectodomain affects binding to its cognate ligand KACL

**Fig 1:**
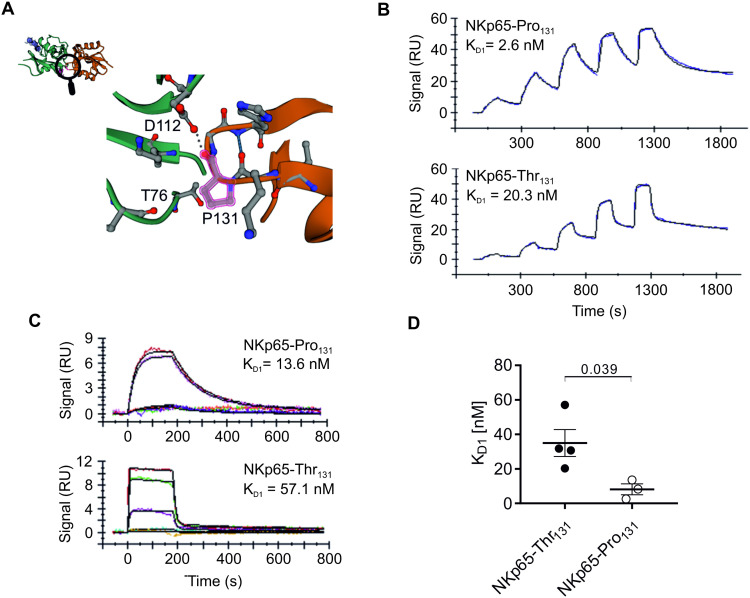
A polymorphism in NKp65 has a significant impact on KACL binding. A, Binding of NKp65 (orange) to KACL (green) [9]. Amino acid 131 (highlighted) is affected by single nucleotide polymorphism resulting in presence of either proline or threonine. Figure was modeled based on the published NKp65-KACL complex structure (PDB: 4IOP) [9]. B, and C, surface plasmon resonance spectroscopy of soluble KACL binding to immobilized NKp65 with either proline (top) or threonine (bottom) at position 131. B, Single-cycle kinetics of sKACL binding to imNKp65. 73.4 ± 12.4 response units (RU) of biotinylated NKp65 were immobilized on a streptavidin sensor chip and pulsed for 120 s with increasing concentrations of sKACL (0.247, 0.741, 2.22, 6.67 and 20 nM). Final dissociation time was set to 600 s**.** Measurements of NKp65 containing proline (top) or threonine (bottom) are shown in blue and were fitted using a bivalent binding model (black). C, Multi-cycle kinetics of sKACL binding to imNKp65. At the top, 22.3 ± 6.2 RU of biotinylated NKp65 (P131) were immobilized on a streptavidin sensor chip and pulsed for 180 s with increasing concentrations of sKACL (0.247, 0.741, 2.22, 6.67 and 20 nM). At the bottom, 29.1 ± 4.8 RU of biotinylated NKp65 (T131) were immobilized on a streptavidin sensor chip and pulsed for 180 s with increasing concentrations of sKACL (9.38, 18.75, 37.5, 75 and 150 nM). Final dissociation time was set to 600 s. Measurements of NKp65 containing proline (top) or threonine (bottom) are shown in color and were fitted using a bivalent binding model (black). D, Mean ± SEM of K_D1_ from at least three measurements is depicted. Recombinant protein was produced independently twice and measured at least 3 times on 2 different chip lots. Statistic was conducted using a two-tailed unpaired Student’s t-test. p = 0.0386; t = 2.786 df = 5; F test to compare variances: F = 8.044, DFn = 3, Dfd = 2, p = 0.2252.

By inspecting the crystal structure of NKp65 bound to KACL [9], we reasoned that the prevalent polymorphism rs576601 in NKp65 (*https://www.ncbi.nlm.nih.gov/snp/*) is likely to affect receptor-ligand interaction. This single nucleotide polymorphism (SNP) locates to amino acid position 131, which is situated in the NKp65-KACL interface and encodes either for threonine or proline (Fig 1A). Though there is some variation among different populations, the NKp65-Thr_131_ variant is more prevalent (68%) on a global scale (S2 Table). To test our hypothesis that polymorphism rs576601 affects NKp65-KACL interaction, we produced recombinant NKp65 ectodomains that contain either proline (NKp65-Pro_131_) or threonine at position 131 (NKp65-Thr_131_) as well as recombinant KACL ectodomains (sKACL) and performed surface plasmon resonance (SPR) measurements. To ensure an orientation that reflects membrane-bound NKp65, we biotinylated the NKp65 ectodomains specifically at the N-terminus via a Bir-A tag and immobilized the ligands to the surface of streptavidin-coated chips. First, we performed single cycle kinetics where immobilized NKp65 was pulsed with increasing concentrations of recombinant KACL (Fig 1B). Binding of the NKp65-Pro_131_ variant showed a high affinity (2.6 nM) for KACL as previously reported [1,9]. Association of KACL with NKp65-Thr_131_ was comparable to that with NKp65-Pro_131_. However, the NKp65-Thr_131_-KACL complex dissociated considerably faster, resulting in a calculated affinity of 20.3 nM (Fig 1B). Next, we performed multi-cycle kinetics with low response units (RU) of NKp65 ligands (Fig 1C) to reach steady state. We increased the analyte concentration from 20 nM to 150 nM to achieve proper binding of KACL to NKp65-Thr_131_. Again, KACL binding towards NKp65-Pro_131_ was of higher affinity than to NKp65-Thr_131_. Together, we confirmed our hypothesis that polymorphism rs576601 in NKp65 affects KACL binding leading to dissociation equilibrium constants of 35.0 ± 7.8 nM for NKp65-Thr_131_ and 8.2 ± 3.2 nM for NKp65-Pro_131_ (Fig 1D) which represents a ~ 4-fold difference (p = 0.039).

Recently, we had introduced the NKp65-specific mAb OMAR1 [5], which was produced by immunizing mice with P815 cells expressing the NKp65-Pro_131_ variant. Since we had observed interference of OMAR1 with KACL binding, we wanted to rule out that polymorphism rs576601 affects OMAR1 binding. In addition, we here introduce a second NKp65-specific mAb called OMAR2 that binds NKp65 via a different epitope non-overlapping with OMAR1 or KACL binding to NKp65. Multi-cycle kinetic analysis of OMAR1 binding to NKp65 (S3 Fig) yielded dissociation equilibrium constants of 0.50 ± 0.15 pM for NKp65-Thr_131_ and 0.48 ± 0.08 pM for NKp65-Pro_131_, respectively. During multi-cycle kinetic measurements, we hardly observed any dissociation of the OMAR1-NKp65 complex.

OMAR2 affinity towards NKp65, however, was in the nanomolar range (S3 Fig) yielding dissociation equilibrium constants of 17.7 ± 3 nM for NKp65-Thr_131_ and 17.5 ± 3.6 nM for NKp65-Pro_131_, respectively. Together, our findings demonstrate that both OMAR1 and OMAR2 bind NKp65 with remarkably high affinity independently from rs576601 and thus are suitable tools to further address this polymorphism, e.g., by ELISA and flow cytometry.

### Reduced surface expression and intracellular retention of NKp65 containing threonine at position 131

**Fig 2:**
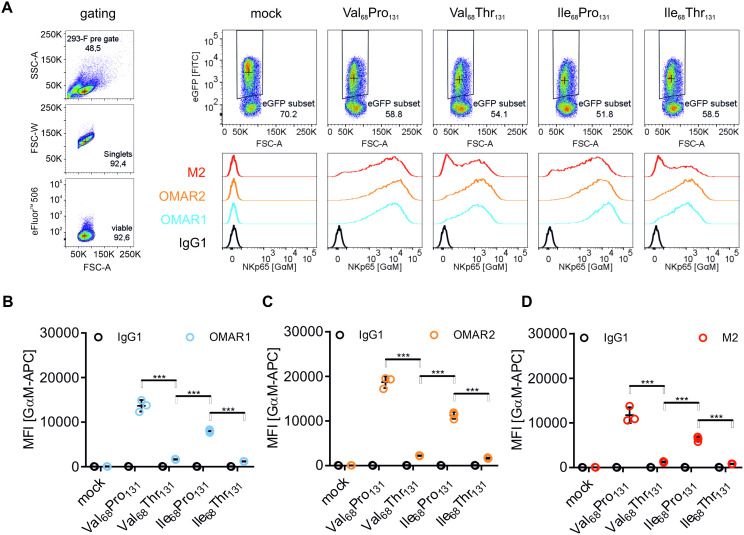
Strongly reduced surface expression of NKp65 containing threonine at amino acid position 131. A, Representative histograms of NKp65 expression on 293-F cells 72 h post transfection with pIRES2-eGFP vector containing cDNA encoding for NKp65 with presence of combination of either proline (Pro) or threonine (Thr) at position 131, isoleucine (Ile) or valine (Val) at position 68 and a C-terminal FLAG tag. Left, 293-F cells were pre-gated according to their size in a FSC-A – SSC-A dot plot. Duplicates and dead cells were excluded from analysis by plotting FSC-A against FSC-W or by FVD eFluor™ 506 staining and gating on negative cells, respectively. Top, transfected cells appeared as eGFP reporter gene positive events. Bottom, eGFP+ transfectants were analyzed for NKp65 expression by primary staining with the anti-NKp65 mAb OMAR1 (blue) and OMAR2 (orange) or the anti-FLAG mAb M2 (red) and secondary staining with APC-conjugated goat anti-mouse antibody. B, C, and D, The MFI (NKp65) of eGFP+ cells stained with OMAR1 (B), OMAR2 (C) or M2 (D) is shown 72 h post transfection. Displayed are single values and means ± SD of three independent experiments. Mock-transfected cells and primary staining with IgG1 (black) mAb served as control, respectively. Statistic was conducted using a one-way ANOVA, considering only the antibody-specific signal and not the isotype control. B, Equality of group variances was tested using a Brown-Forsythe test: F (DFn, DFd) = 3.01 (4, 10), p = 0.0718 considering SDs as not significantly different. ANOVA summary: F = 271.2, p < 0.0001, R2 = 0.9909, Tukey’s multiple comparison test was conducted to compare column means, significance is indicated by *** (p < 0.001), n = 3. C, Equality of group variances was tested using a Brown-Forsythe test: F (DFn, DFd) = 0.9459 (4, 10), p = 0.4769 considering SDs as not significantly different. ANOVA summary: F = 406.8, p < 0.0001, R2 = 0.9939, Tukey’s multiple comparison test was conducted to compare column means, significance is indicated by *** (p < 0.001), n = 3. D, Equality of group variances was tested using a Brown-Forsythe test: F (DFn, DFd) = 1.133 (4, 10), p = 0.3950 considering SDs as not significantly different. ANOVA summary: F = 107.3, p < 0.0001, R2 = 0.9772, Tukey’s multiple comparison test was conducted to compare column means, significance is indicated by *** (p < 0.001), n = 3.

A second polymorphism occurs with relevant frequency (S4 Table) in the coding sequence of NKp65 and gained our interest: rs1797517 locates to the stalk region at amino acid position 68 within an N-linked glycosylation motif (NxS). This polymorphism encodes for either valine (24%) or isoleucine (76%). Hence, together with rs576601, there are four possible amino acid combinations. Here, we tested the impact of polymorphisms rs1797517 and rs576601 on NKp65 surface expression as well as NKp65 glycosylation. For this purpose, 293-F transfectants were stained with the α-NKp65 mAbs OMAR1 (Fig 2B) and OMAR2 (Fig 2C), or with the α-FLAG tag Ab M2 (Fig 2D) binding NKp65 at its FLAG-tagged C-terminus. Our data suggest that Pro_131_ favors NKp65 surface expression, as we observed significantly increased mean fluorescence intensities (MFI) of NKp65 transfectants containing Pro_131_ (Fig 2B-D). Similar transfection efficiencies were assured by monitoring enhanced green fluorescent protein (eGFP) expression (Fig 2A).

**Fig 3:**
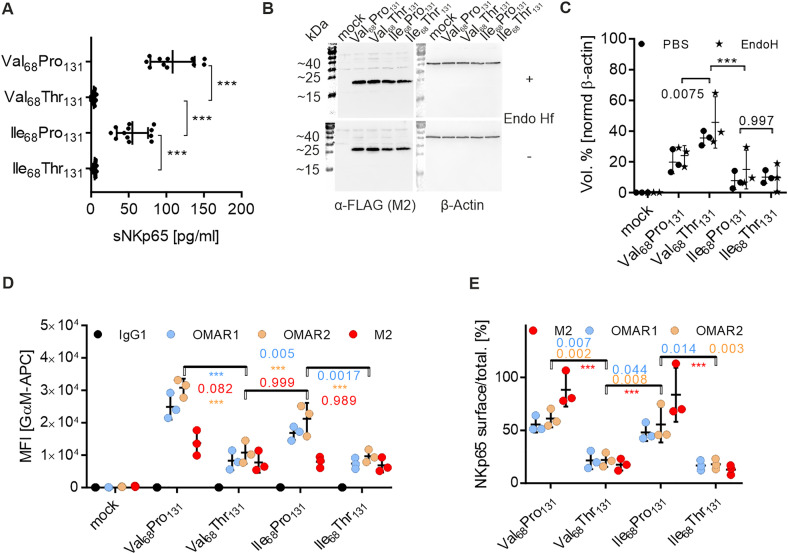
Intracellular retention, but not shedding of NKp65 molecules containing threonine at amino acid position 131 causes decreased surface expression. A, concentration of soluble NKp65 (sNKp65) in the culture supernatant of 293-F transfectants 72 h post transfection. Determined by sandwich ELISA using OMAR2 as capture and OMAR1-bio as sandwich antibody. Depicted are quadruplicates of three independent experiments as well as overall means ± SD. Statistic was conducted using a one-way ANOVA. Equality of group variances was tested using a Brown-Forsythe test: F (DFn, DFd) = 11.02 (3, 43), p < 0.0001 considering SDs as significantly different. Unequal variances were confirmed by Bartlett’s test: 74.58, p < 0.0001. ANOVA summary: F = 90.1, p < 0.0001, R2 = 0.8627, Tukey’s multiple comparison test was conducted to compare column means, significance is indicated by *** (p < 0.001), n = 3 with quadruplicates. B, representative immunoblot of Endo Hf-treated (+) and PBS-treated (-) whole cell lysate using β-actin-HRP directly conjugated antibody (right) or α-M2/GαM-HRP antibodies to stain for FLAG-tagged NKp65 (left). 20 µg of total protein were loaded for each sample. C, Signal intensities of NKp65 immunoblots were quantified using Fusion’s analysis tool. The FLAG-tag specific signals were normalized to respective β-actin signals (Vol. % [normd β-actin]). Displayed are single values and means ± SD of three independent experiments. Statistic was conducted using two-way ANOVA. Row factor (polymorphism) accounts for 77.61% of total variation with F (4, 20) = 20.54 and p < 0.0001. Column Factor (Endo Hf) with F (1, 20) = 1.915 and p = 0.1816 was not considered to be significant and no interaction of the factors was observed (p = 0.7742). The main row effect was analyzed using Tukey’s multiple comparison test, *** equates p < 0.001. D, staining of permeabilized 293-F transfectants for both intracellular and surface NKp65 with M2, OMAR1 and OMAR2 antibodies. Shown are single values and means ± SD of three independent experiments. Statistic was conducted using a two-way ANOVA, excluding the IgG1 control staining. The row factor (polymorphism) accounts for 73.04% of total variation and was considered as highly significant: F (8, 30) = 89.28, p < 0.0001. The column factor (antibody) accounts for 10.85% of variation and was considered as highly significant, as well: F (4, 30) = 26.52, p < 0.0001. Interaction of both factors accounts for 9.972% of variation and was significant as well, however with a low F value of (8, 30) 6.095, p = 0.0001. Within each column, the rows were compared (simple effects within columns) using a Tukey’s multiple comparison test, alpha was set to 0.05 and one family per column was created. The p-values are shown in the graph using the same color coding as for antibodies. E, NKp65 surface expression data of 293-F cells from Fig 2 was set in ratio to whole cell signal of 293-F cells from Fig 3D. Displayed are single values and means ± SD of three independent experiments. Statistic was conducted as described in D, row factor (polymorphism) accounts for 73.82% of total variation, F (3, 24) = 51.26, p < 0.0001. Column factor (antibody) accounts for 5.417% of variation, with a low F-value of (2, 24) 5.641, p = 0.01. Interaction accounts for 9.236% of variation with a weak F-value of (6, 24) 3.206, p = 0.02. The p-values of Tukey’s multiple comparison are shown in the graph using the same color coding as for antibodies.

Next, we used OMAR1 and OMAR2 in a sandwich ELISA, to test for shed NKp65 molecules in culture supernatants (Fig 3A). The concentration of soluble NKp65 was proportional to NKp65 surface expression of the respective transfectants. We therefore exclude any impact of the two polymorphism on shedding of membrane-bound NKp65. As neither transfection efficiency nor soluble NKp65 from culture supernatant could explain the reduced surface expression of the two NKp65-Thr_131_ variants, we hypothesized that these are retained intracellularly. To test our hypothesis, immunoblotting of whole cell lysates of the transfectants was performed (Fig 3B). NKp65-specific signal (by detecting the FLAG-tag) was quantified by normalization to actin, followed by global normalization to the NKp65-Val_68_Pro_131_ signal (Fig 3C). In combination with valine at position 68, we observed a moderately increased signal for the NKp65-Thr_131_ variant as compared to the respective NKp65-Pro_131_ variant. The slightly reduced surface expression of the NKp65 construct bearing both isoleucine and proline (Fig 2) was not explained by intracellular retention but rather a consequence of a lower total abundance, an effect that was generally observed for both constructs containing isoleucine (Fig 3C). Essentially, neither in combination with valine nor isoleucine, the increased surface expression of NKp65-Pro_131_ molecules could be explained by increased total protein levels, supporting our hypothesis of an intracellular retention of the threonine variants. Of note, all detectable NKp65 molecules from whole cell lysates were sensitive to treatment with Endo Hf (Fig 3B). As we observed strong surface expression in flow cytometry, but no doublets in immunoblot, we reasoned that NKp65 in 293-F transfectants do not contain complex glycan structures but more likely high mannose or hybrid structures, which are susceptible to Endo Hf digestion [16]. To further address our hypothesis about an intracellular retention of NKp65-Thr_131_ molecules, flow cytometric analyses of NKp65 surface expression (Fig 2) was complemented by flow cytometric analyses of permeabilized 293-F transfectants including both, surface and intracellular NKp65 molecules (Fig 3D). Subsequently, the MFIs of permeabilized cells were interrelated with the corresponding NKp65 surface stainings, resulting in a calculated percentage surface expression (Fig 3E). Accordingly, whereas the NKp65-Thr_131_ variants were barely expressed at the surface (18.1 ± 5.87%), roughly two-thirds (65.3 ± 20.1%) of all NKp65-Pro_131_ molecules were exposed at the cell surface. The results further support an intracellular retention of NKp65-Thr_131_ as compared to NKp65-Pro_131_.

Together, we here show that polymorphism rs576601 significantly affects NKp65 surface expression by intracellular retention of the NKp65-Thr_131_ variants.

### Proline as compared to threonine at position 131 in NKp65 favors killing of KACL-expressing target cells

**Fig 4:**
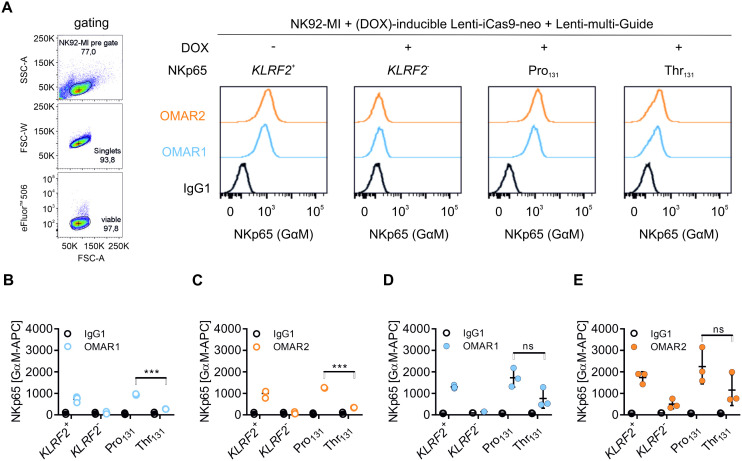
Establishment of NK92-MI effector cell lines expressing NKp65 variants by KLRF2 knockout using an inducible CRISPR system (iCas9) and lentiviral reconstitution. Stable NK92-MI cell lines deficient in endogenous NKp65 expression (*KLRF2*^*-*^) were generated by infection with lentiviruses containing the (DOX)-inducible CRISPR/Cas9 system (Lenti-iCas9-neo) as well as sgRNA targeting KLRF2 (Lenti-multi-Guide). NKp65 variants of polymorphism rs576601 (Pro/Thr131) were reconstituted by retroviral transduction (pMXs-IP). A, gating strategy (left: FSC-A vs. SSC-A, FSC-A vs. FSC-W, FSC-A vs. AmCyan-A) and representative histograms of NKp65 surface expression (right) of transduced NK92-MI cells that were treated with DOX (1 µg/ml). Data were assessed by flow cytometry upon staining of cells with fixable viability dye eFluor™ 506, primary mAb OMAR1 and OMAR2, respectively (IgG1 control), as well as APC-conjugated secondary goat-anti-mouse antibody. B, and C, NKp65 surface expression of NK92-MI effector cell lines. Shown are means ± SD of three independent stainings as described in A. D, and E, NKp65 expression of permeabilized NK92-MI effector cell lines. Shown are means ± SD of three independent stainings as described in A. Significance was tested using either a Student’s (B and C) or a Welch’s (D and E) t-test. N = 3; *** indicates a p-value <0.001, ns means not significant.

**Fig 5:**
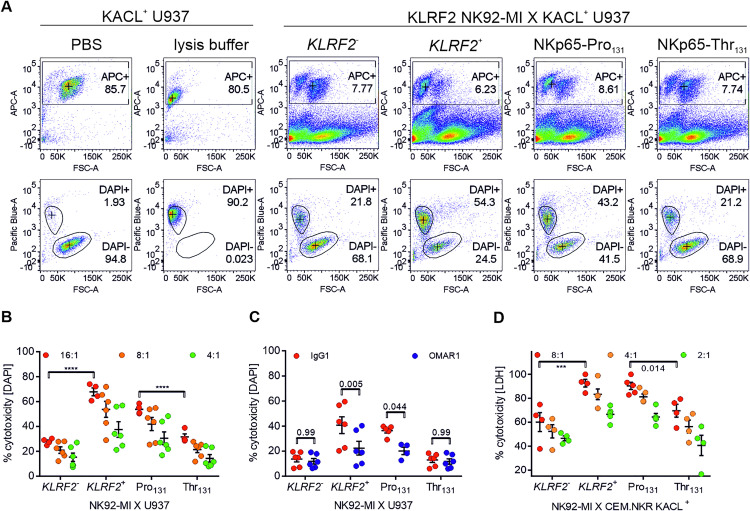
Proline 131 in NKp65 favors killing of KACL-expressing target cells as compared to threonine 131. A, Representative pseudocolor plots of FarRed™ labeled KACL^+^ U937 target cells and U937 co-cultured with various NKp65-expressing NK92-MI cell lines (E:T = 8:1). The FarRed™ (APC) signal is plotted against FSC-A (x-axis). APC^+^ target cells were gated and analyzed for viability by DAPI signal (Pacific Blue). B, NK92-MI NKp65^+^ effector cells were co-cultured with KACL^+^ FarRed-labeled target cells at different E:T ratios (indicated by color) for 4 h. Shown are individual values and means ± SEM. N = 4 (16:1) and n = 6 (8:1 and 4:1), 2way ANOVA with Tukey’s multiple comparison test (alpha = 0.05) was conducted to interpret simple effect within columns (E:T ratio). Cell line (row factor) accounts for 53.09% of total variation, F (DFn, DFd) = 36.01 (3, 52), p < 0.0001. C, Cellular cytotoxicity of NK92-MI effector cells towards U937 target cells after 4 h at an E:T ratio of 8:1 in the presence of the blocking mAb OMAR1 (blue) or of an IgG1 isotype control (red). N = 6, Depicted are single values and means ± SEM. Statistic was conducted using 2way ANOVA and Sidak’s multiple comparison test to compare each cell mean (OMAR1) with the other cell mean (IgG1) in that row (cell line). Interaction for row (cell line) and column factor (blocking) was significant, F (DFn, DFd) = 2.9 (3, 37), p = 0.048. D, Cellular cytotoxicity of NK92-MI effector cells towards CEM.NKR-KACL target cells after 4 h of co-culture at an E:T of 8:1, 4:1 and 2:1, measured by an LDH release assay. Depicted are single values and means ± SEM. Statistic was conducted using 2way ANOVA and Tukey’s multiple comparison test to analyze simple effects within columns (E:T ratio). Cell line (row factor) accounts for 48.8% of total variation, F (DFn, DFd) = 30.24 (3, 41), p < 0.0001. E:T ratio (column factor) accounts for 27.9% of variation, F (DFn, DFd) = 25.94 (2, 41), p < 0.0001. No interaction of both factors was observed, F (DFn, DFd) = 0.585 (6, 41), p = 0.74.

Given the significantly reduced affinity and surface expression of the NKp65-Thr_131_ variant, we wondered whether this polymorphism affects cellular cytotoxicity towards KACL-expressing target cells. To address this question, endogenous NKp65 expression in NK92-MI cells was eliminated by doxycycline (DOX)-inducible clustered regularly interspaced short palindromic repeats (CRISPR)/Cas9 according to Cao et al. [14], before reconstitution of NKp65 expression by lentiviral transduction of constructs containing either NKp65-Pro_131_ or NKp65-Thr_131_ (Fig 4). Of note, cell surface expression of NKp65 was also reduced on NK92-MI NKp65-Thr_131_ transductants, suggesting an intrinsic effect of the rs576601 polymorphism independently of the cell type (Fig 4). Since U937 cells endogenously express the NKp65 ligand KACL, they were chosen as target cells for the killing assay [1]. U937 cells were labelled with FarRed fluorescent dye prior to 4 h incubation with NKp65-expressing NK92-MI effector cells. Upon DAPI staining, we could distinguish between two cell subsets by flow cytometry within the FarRed^+^ (APC) subset (Fig 5A). Percent cytotoxicity was calculated relative to U937 single cultures after subtraction of spontaneous target cell death, yielding 53.8% cytotoxicity for NKp65-Pro_131_ and 31.6% cytotoxicity for NKp65-Thr_131_ at an E:T of 16:1 (Fig 5B). Of note, the cytotoxicity of NKp65-negative NK92-MI cells towards U937 cells already was at ~28%. This shows that the NKp65-Thr_131_ variant barely triggered cytotoxicity above background levels and that there are other cytotoxic interactions besides the NKp65-KACL axis. Moreover, pre-incubation of effector cells with OMAR1 significantly reduced cytotoxicity of *KLRF2*^*+*^ NK92-MI and NKp65-Pro_131_ NK92-MI cells demonstrating the cytotoxic effect of the NKp65-KACL axis (Fig 5C). To exclude the possibility that the observed differential NKp65-mediated cytotoxicity was peculiar for the NK92-MI/U937 co-culture system, we also addressed cytotoxicity towards CEM.NKR cells, which were transduced to express KACL [17], using a LDH release assay (Fig 5D and S5 Fig). While all NK92-MI cell lines showed comparable cytotoxicity towards mock-transduced CEM.NKR mock cells (S5 Fig), the cytotoxicity towards CEM.NKR KACL transductants was significantly enhanced for both, *KLRF2*^*+*^ and NKp65-Pro_131_ effector cells as compared to *KLRF2*^*-*^ and NKp65-Thr_131_ NK92-MI cell lines (Fig 5D).

**Fig 6:**
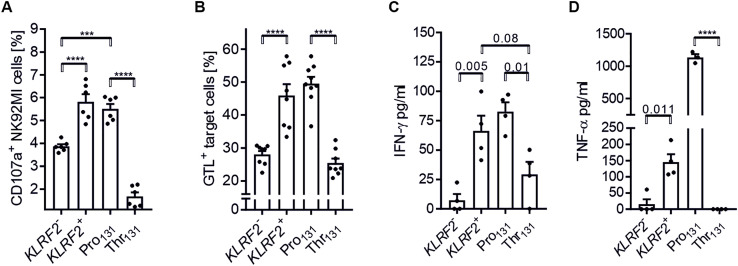
NKp65 polymorphism rs576601 affects functional responses towards KACL-expressing target cells. A, Degranulation (CD107a) of NK92-MI cells in co-culture with U937 cells displayed as percentages of effector cells using flow cytometry and PE-conjugated anti-CD107a antibody. Shown are single values and mean ± SEM of n = 3 experiments with technical duplicates. Statistic was conducted using one-way ANOVA: F = 73.02, p < 0.0001, R square = 0.9163. A Brown-Forsythe test (F (DFn, DFd) = 9.588 (3, 20)) and a Bartlett’s test (6.211), p = 0.10 were conducted to compare group variances. A Tukey’s multiple comparison test with alpha = 0.05 was used to compare group means. B, Granzyme B delivery of NK92-MI cells towards U937 target cells was quantified using the GranToxiLux® assay kit and flow cytometry. Shown are the percentages of GTL-positive target cells. Displayed are single values and means ± SEM of n = 3 experiments with triplicates (n = 2) and duplicates (n = 1). Statistic was conducted using one-way ANOVA: F = 31.99, p < 0.0001, R square = 0.768. A Brown-Forsythe test (F (DFn, DFd) = 4.164 (3, 29)) and a Bartlett’s test (9.967), p = 0.019 were conducted to compare group variances. A Tukey’s multiple comparison test with alpha = 0.05 was used to compare group means. C, IFN-γ secretion by NK92-MI cells upon CEM.NKR-KACL co-culture. Concentration was determined by ELISA. Depicted are means of technical triplicates of n = 4 experiments as well as overall mean ± SEM. Statistic was conducted using one-way ANOVA: F = 12.65, p = 0.0005, R square = 0.7597. A Brown-Forsythe test (F (DFn, DFd) = 0.9206 (3, 12), p = 0.46) was conducted to compare group variances. A Tukey’s multiple comparison test with alpha = 0.05 was used to compare group means. D, TNF-α secretion by NK92-MI cells upon CEM.NKR-KACL co-culture. Concentration was determined by ELISA. Depicted are means of technical triplicates of n = 4 experiments as well as overall mean ± SEM. Statistic was conducted using one-way ANOVA: F = 426.6, p < 0.0001, R square = 0.9915. A Brown-Forsythe test (F (DFn, DFd) = 2.194 (3, 11), p = 0.146) was conducted to compare group variances. A Tukey’s multiple comparison test with alpha = 0.05 was used to compare group means.

This differential NKp65-mediated cytotoxic response of NKp65-Thr_131_ versus NKp65-Pro_131_ expressing NK92-MI cell lines was further corroborated by assessing degranulation of the NK92-MI cell lines in co-cultures with U937 cells (Fig 6A and S6 Fig). In addition, we made use of the GranToxiLux® STAR protocol [18] to assess NKp65-mediated cytotoxicity. In alignment with the other cytotoxicity assays, we found that Granzyme B delivery to U937 target cells was much more pronounced for NKp65-Pro_131_ NK92-MI cells as compared to NKp65-Thr_131_ NK92-MI cells (Fig 6B and S7 Fig). Finally, we addressed an impact of the NKp65 polymorphism rs576601 on cytokine secretion. To this aim, the NKp65-expressing NK92-MI cell lines were co-cultured with CEM.NKR-KACL cells and supernatants analyzed for IFN-γ (Fig 6C) and TNF-α (Fig 6D). *KLRF2*^*+*^ and NKp65-Pro_131_ NK92-MI cells secreted significantly more IFN-γ and TNF-α upon co-culture with KACL^+^ CEM.NKR cells as compared to *KLRF2*^*-*^ and NKp65-Thr_131_ NK92-MI cells.

Collectively, our data show that polymorphism rs576601 drastically affects functional interaction of NKp65^+^ effector cells with KACL^+^ target cells.

## Discussion

The genetically-coupled C-type lectin-like receptors NKp65 and KACL represent a high affinity receptor-ligand pair [1]. Very recently, we reported that NKp65 is selectively expressed by human ILC3 and demarcates human ILC3 from NK cells, which express the NKp65 sibling receptor NKp80 [5]. Since the NKp65 ligand KACL is almost exclusively expressed on human keratinocytes, we propose that the NKp65-KACL axis specifically mediates immunosurveillance of human skin by ILC3.

Searching of the SNP database revealed that there are two common coding single nucleotide polymorphism in the NKp65 ectodomain, isoleucine versus valine at position 68 (rs1797517) as well as proline versus threonine at position 131 (rs576601). Since the latter is positioned in the interface between NKp65 and KACL, we postulated that this polymorphism affects the interaction of NKp65 with KACL. However, only the NKp65-Pro_131_ variant has previously been subjected to affinity measurements [1,9], although the NKp65-Thr_131_ variant is more prevalent in the population. In fact, our interaction studies using surface plasmon resonance revealed that the NKp65-Thr_131_ variant exhibits a ~ 4-fold lower affinity (35 nM) towards its cognate ligand KACL as compared to the NKp65-Pro_131_ variant (~8 nM). The latter is in accordance with the earlier measurements of the NKp65-Pro_131_-KACL affinity [1,9]. Based on the NKp65-KACL crystal structure, substitution of Pro_131_ by Thr_131_ likely affects a hydrogen bond to KACL Thr_76_ as well as van der Waals contacts to KACL Thr_76_ and Asp_112_ [9]. Ultimately, this needs to be clarified by determining the structure of the NKp65-Thr_131_-KACL complex.

We recently introduced the NKp65-specific mAb OMAR1 [5]. Here, we present OMAR2 as a second NKp65-specific mAb that binds NKp65 through a different epitope non-overlapping with OMAR1 or KACL binding to NKp65. In this study, we rule out that polymorphism rs576601 affects OMAR1 and OMAR2 binding. Moreover, using both antibodies we introduce a suitable antibody-system to detect soluble NKp65 by an NKp65-specific sandwich ELISA.

Flow cytometry studies of 293F cells transfected with FLAG-tagged NKp65 constructs revealed that polymorphism rs576601 not only affects the affinity of the NKp65-KACL complex, but also regulates NKp65 surface expression. NKp65 variants containing Thr_131_ showed a drastically reduced surface expression as compared to NKp65-Pro_131_ variants. Immunoblotting demonstrated that substitution of Pro_131_ by Thr_131_ results not only in a significantly reduced surface expression but also an about two-fold increase of intracellular NKp65, suggesting the NKp65-Thr_131_ variants are intracellularly retained. Similar effects of a polymorphism on surface expression and intracellular retention were previously described for the inhibitory killer-cell Ig-like receptor CD158f [19]. Further studies, e.g., by confocal microscopy, may shed light on the localization of intracellularly retained NKp65. Of note, NKp65 molecules were sensitive to Endo Hf treatment including NKp65-Pro_131_, which is strongly expressed at the cell surface. N-glycan processing in the Golgi leads to three classes of glycans [20]. The Endo Hf-sensitive high-mannose and hybrid glycans as well as Endo Hf-resistant complex glycans [16]. Accordingly, we exclude complex glycosylation of NKp65 molecules in 293F transfectants. Moreover, we can exclude glycosylation being responsible for intracellular retention as both, NKp65-Pro_131_ and NKp65-Thr_131_, are deglycosylated in a similar manner. Further experiments are necessary to address the localization, and possibly function, of intracellularly retained NKp65-Thr_131_ molecules.

Finally, we show that the polymorphism rs576601 of NKp65 strongly affects the function mediated by the NKp65-KACL axis. While KACL-positive U937 and CEM.NKR cells were clearly killed by NK92-MI cells bearing the NKp65-Pro_131_ variant, the cytolytic capacity of NK92-MI cells expressing the NKp65-Thr_131_ variant towards these cell lines was substantially lower, and was similar to the cytolytic capacity of *KLRF2* knockout NK92-MI cells that do not express surface NKp65. This likely can be explained by a combination of reduced expression of NKp65-Thr_131_ variants and a lower affinity of the NKp65-Thr_131_-KACL complex.

Recently, Magnaldo and colleagues described an antitumoral involvement of the NKp65-KACL axis, namely the restriction of squamous cell carcinoma invasion in *Xeroderma pigmentosum* patients [7] which led to the notification of the *KLRF2* gene in the DISGENET database [21]. Even though they attributed NKp65-expression to NK cells, which is in disagreement with our more recent publication demonstrating NKp65 as an ILC3 marker [5], the NKp65-KACL axis is likely involved in immunosurveillance of the skin.

To our knowledge, none of the abovementioned polymorphisms were addressed thus far with regard to their clinical relevance and they are not yet listed in the National Center for Biotechnology Information (NCBI) ClinVar database [22]. Consequently, it will be of great interest to examine the physiological relevance of rs576601 *in vivo* in upcoming studies. As the NKp65-KACL axis likely contributes to the immunosurveillance of human skin by ILC3, investigation of a differential impact of the NKp65-Pro_131_ versus the NKp65-Thr_131_ variant in inflammatory skin diseases, e.g., in psoriatic disease, will be of substantial interest.

## Supporting information

S1 Raw Images Raw images associated with immunoblots in Fig 3C. (PDF)

S2 Table Prevalence of SNP rs576601 in different populations. The triplet XCT determines amino acid 131 of NKp65 that can be either threonine (X = Adenine) or proline (X = Cytosine). Adapted from Reference SNP (rs) Report of rs576601, National Library of Medicine (Status: 9/2022). Ref Allele = Reference Assembly allele; Alt Allele = Alternate allele; Ref HMOZ = Reference Homozygous genotype Frequency; Alt HMOZ = Alternate Homozygous genotype Frequency; HTRZ = Heterozygous genotype Frequency; HWEP = -Log(HWE Probability).
(PDF)

S3 Fig Antibodies OMAR1 and OMAR2 bind NKp65 with high affinity independently from polymorphism rs576601. A and B, surface plasmon resonance spectroscopy multi-cycle kinetic measurement of mAb OMAR1 binding to immobilized NKp65. 49.6 ± 2.8 (A, NKp65-Pro_131_) and 48.1 ± 5.1 (B, NKp65-Thr_131_) RU of NKp65 were immobilized on a streptavidin chip and pulsed for 180 s with soluble mAb OMAR1 (1.25, 2.5, 5, 10 and 20 nM). Dissociation time was set to 600 s. Replicates (color) and bivalent fits (black) of one representative experiment are depicted. C, 45.3 ± 19.5 RU (Thr_131_) and 47.9 ± 20.6 RU (Pro_131_) of NKp65 were immobilized. Single values and mean ± SEM of K_D1_ of OMAR1 binding to NKp65 from two measurements are shown. A two tailed unpaired Student’s t-test was conducted, n = 3, p = 0.881 considered as not significant (ns); t = 0.1602, df = 4; F test to compare variances: F = 3.399, DFn = 2, Dfd = 2, p = 0.455. Recombinant protein was produced independently twice and measured three times on two different chip lots. D and E, surface plasmon resonance spectroscopy multi-cycle kinetic measurement of mAb OMAR2 binding to immobilized NKp65. 71.9 ± 1.8 (D, NKp65-Pro_131_) and 87.5 ± 5.3 (E, NKp65-Thr_131_) RU of NKp65 were immobilized on a streptavidin chip and pulsed for 180 s with soluble mAb OMAR2 (1.25, 2.5, 5, 10 and 20 nM). Dissociation time was set to 600 s. Replicates (color) and bivalent fits (black) of one representative experiment are depicted. F, 89.5 ± 5.4 RU (Thr_131_) and 52 ± 20.7 RU (Pro_131_) of NKp65 were immobilized. Single values and mean ± SEM of K_D1_ of OMAR2 binding to NKp65 are shown. A two tailed unpaired Student’s t-test was conducted, n = 2, p = 0,97 considered as not significant (ns); t = 0.04268, df = 2. Recombinant protein was produced independently twice and measured two times on two different chip lots.
(TIF)

S4 Table Prevalence of SNP rs1797517 in different populations. The triplet XTC determines the amino acid 68 of NKp65 that can be either valine (X = Guanine) or isoleucine (X = Adenine). Adapted from Reference SNP (rs) Report of rs1797517, National Library of Medicine (Status: 9/2022). Ref Allele = Reference Assembly allele; Alt Allele = Alternate allele; Ref HMOZ = Reference Homozygous genotype Frequency; Alt HMOZ = Alternate Homozygous genotype Frequency; HTRZ = Heterozygous genotype Frequency; HWEP = -Log(HWE Probability).
(PDF)

S5 Fig Comparable cytotoxicity of NK92-MI effector cell lines towards CEM. NKR-mock transductants. Cellular cytotoxicity of NK92-MI effector cells towards KACL-negative CEM.NKR-mock transductants after 4 h of co-culture at an E:T of 8:1, 4:1 and 2:1, measured by target cell LDH release. Depicted are single values and means ± SEM. Statistic was conducted using 2way ANOVA and Tukey’s multiple comparison test to analyze simple effects within columns (E:T ratio). Cell line (row factor) accounts for 0.74% of total variation, F (DFn, DFd) = 0.35 (3, 36), p = 0.79 and was considered not significant. E:T ratio (column factor) accounts for 69.52% of variation, F (DFn, DFd) = 49.52 (2, 36), p < 0.0001. No interaction of both factors was observed, F (DFn, DFd) = 1.060.585 (6, 36), p = 0.40.
(TIF)

S6 Fig Degranulation of NK92-MI cells in U937 co-cultures. Effector cells were labeled with FarRed™ prior to single culture (top) or co-culture (bottom) with U937 cells. Subsequently, cells were stained with PE-conjugated anti CD107a antibody. Gate Q1 shows degranulation of viable NK92-MI effector cells. Shown are representative pseudocolor plots. Data are summarized in Fig 6A.
(TIF)

S7 Fig Granzyme B delivery of NK92-MI effector cells towards U937 target cells. Target cells were labeled with TFL4 (APC) and cultured with (right) or without (left) NK92-MI effector cells in the presence of GranToxiLux® substrate for 4 h. Cleavage of GranToxiLux® by Granzyme B was measured in the FITC channel. Shown are representative pseudocolor plots from one of three independent experiments with technical triplicates.
(TIF)
