# Supplementary material for: A common polymorphism in the human immunoreceptor NKp65 determines ligand interaction, cell surface expression and function
Source: PLoS One. 2025 Aug 13;20(8):e0329454. doi: 10.1371/journal.pone.0329454 (PMC12349009; doi:10.1371/journal.pone.0329454)
Supplement: S1 Raw Images — (PDF) [file pone.0329454.s001.pdf]

Immunoblot of whole cell lysate from 293-F transfectants using the FLAG-tag specific antibody M2 and the secondary antibody goat-anti-mouse HRP

Marker

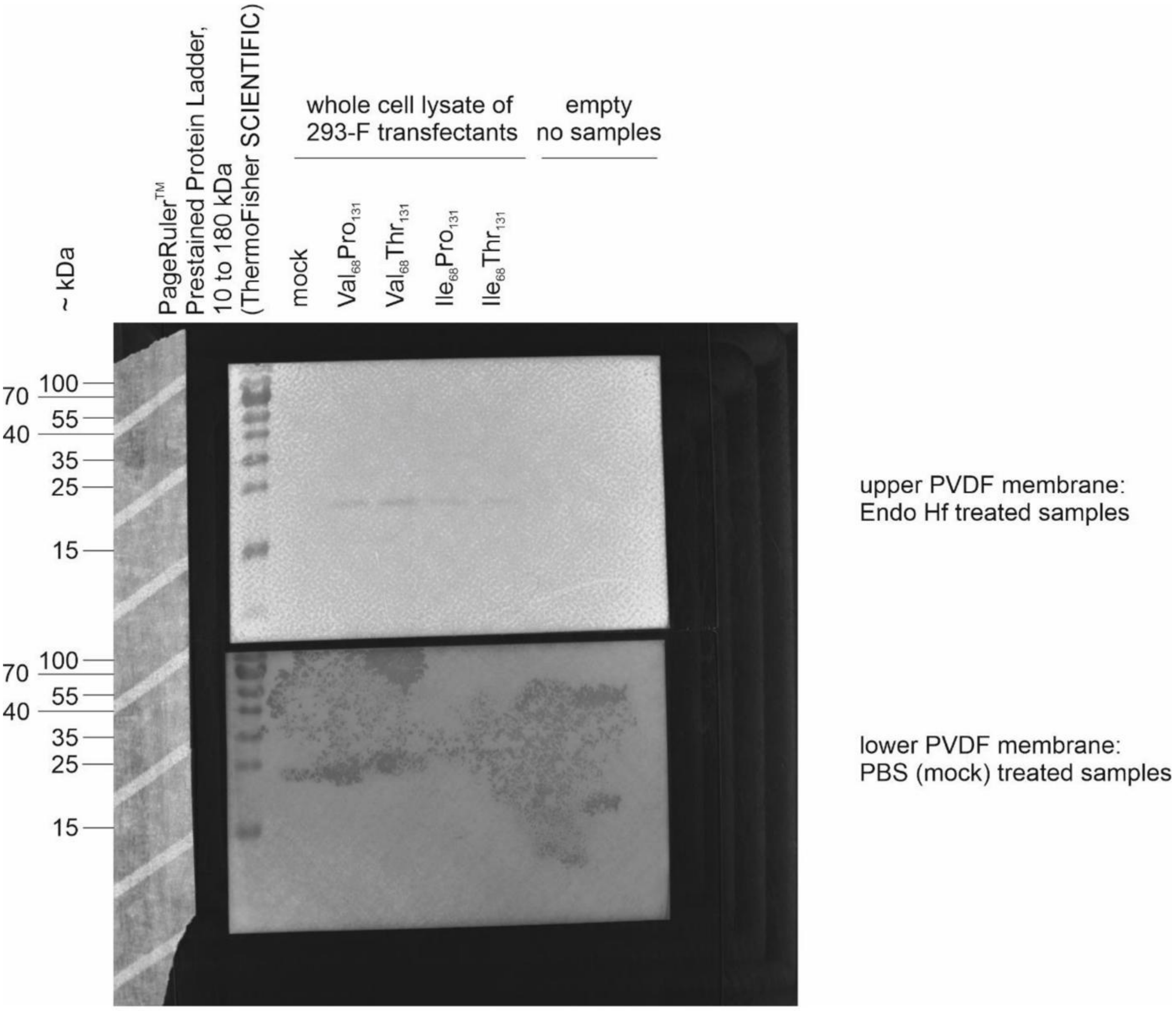

Picture was taken using weak white light in a FUSION SL apparatus (Vilber Lourmat) and the FusionCapt Advance SL4 16.09b software. Shutter was set to max (0.84) for two seconds.

Chemiluminescence

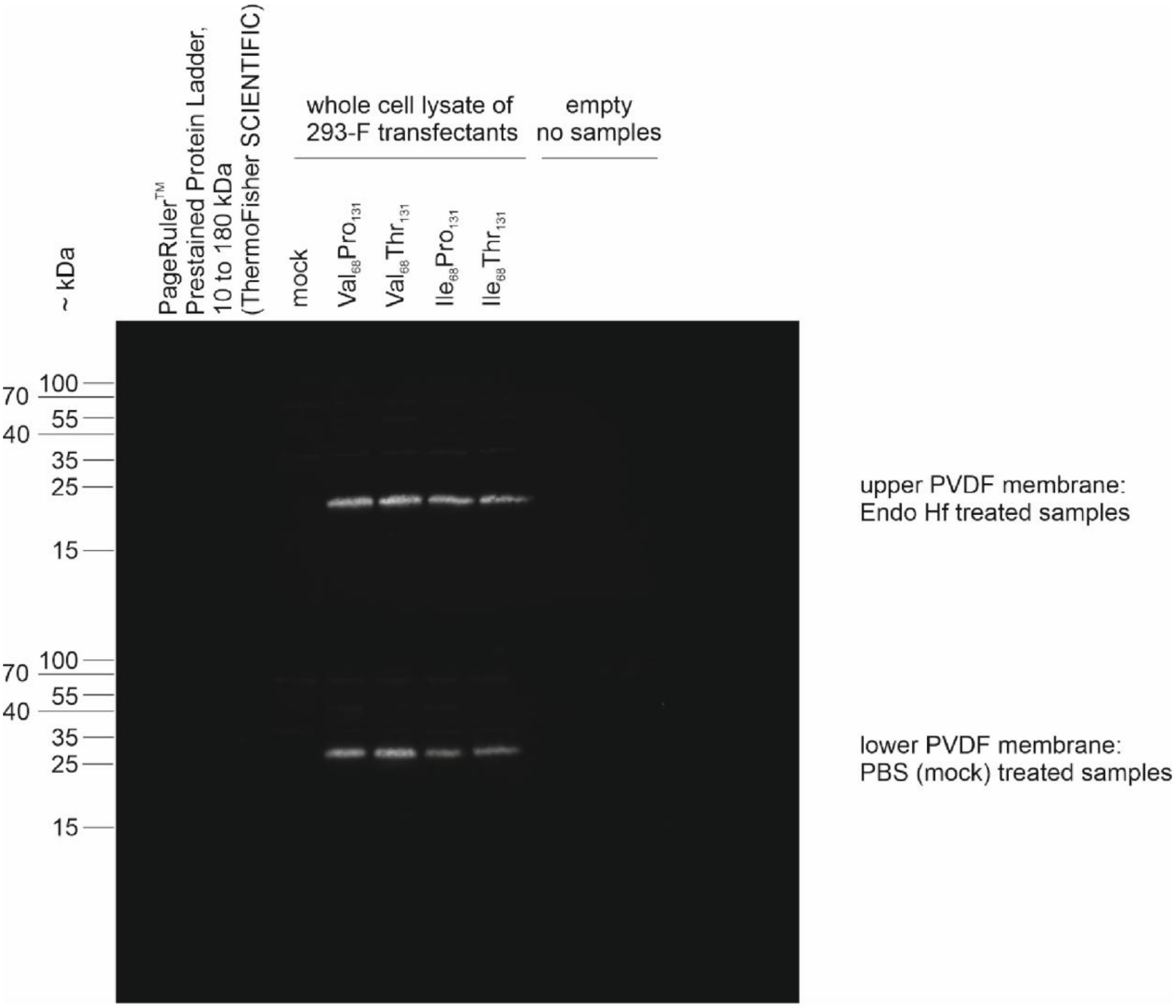

Picture was taken using the chemiluminescence function and the "full resolution" application for quantification in a FUSION SL apparatus (Vilber Lourmat) and the FusionCapt Advance SL4 16.09b software. Shutter was set to max (0.84) for two seconds test exposure. The auto exposure time was set to 2 minutes and 34.1 seconds by the program.

Merge

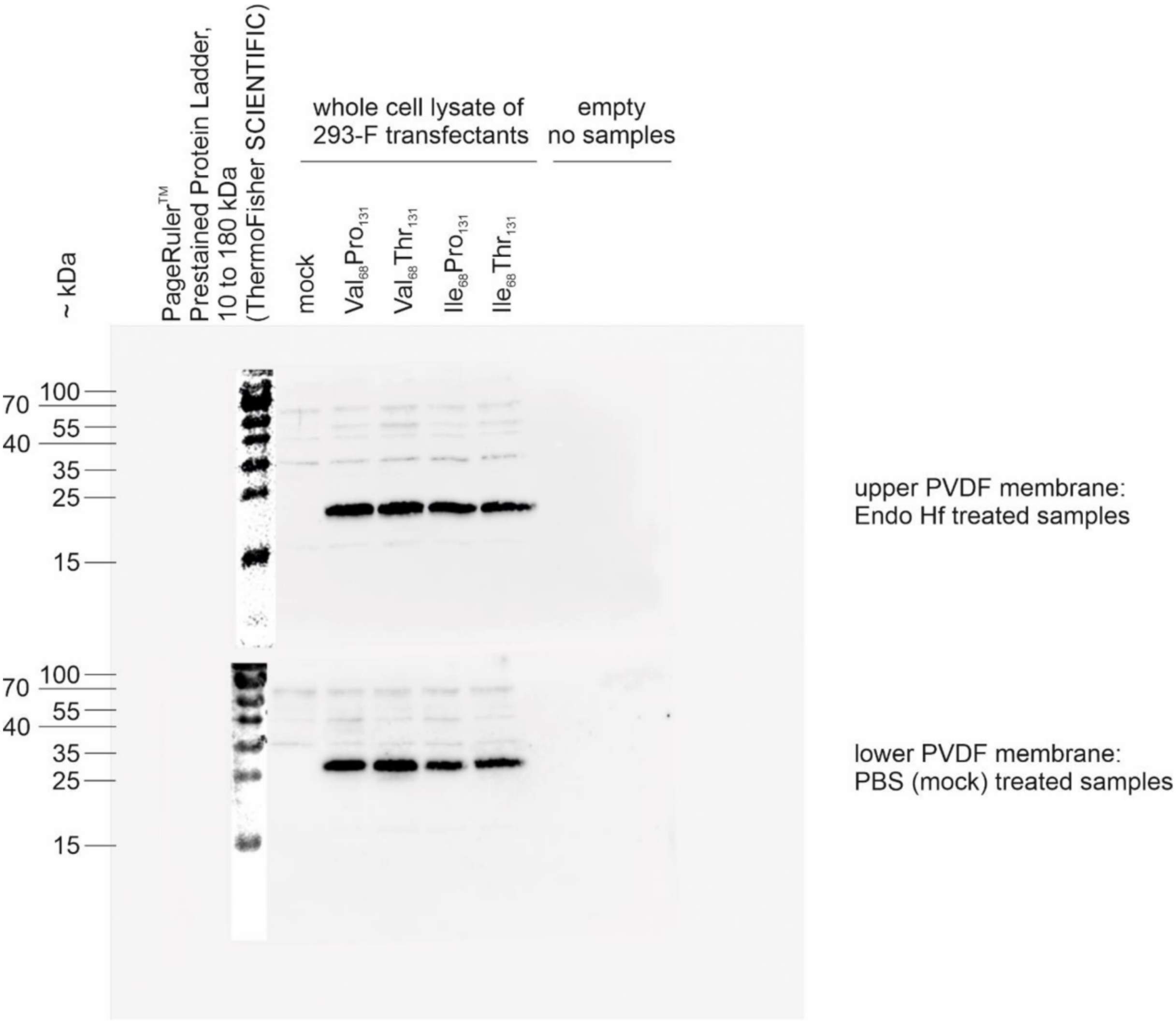

Using the FusionCapt Advance software, the chemiluminescence picture was inverted before the marker was merged using the implemented "multiple image processing" tool.

Next, the membranes were stripped using Re-Blot Plus Mild solution (Merck, Darmstadt, Hessen, Germany) according to manufacturers' instructions.

Immunoblot of whole cell lysate from 293-F transfectants using the stripped membranes from above as well as a HRP-conjugated β-actin antibody

Marker

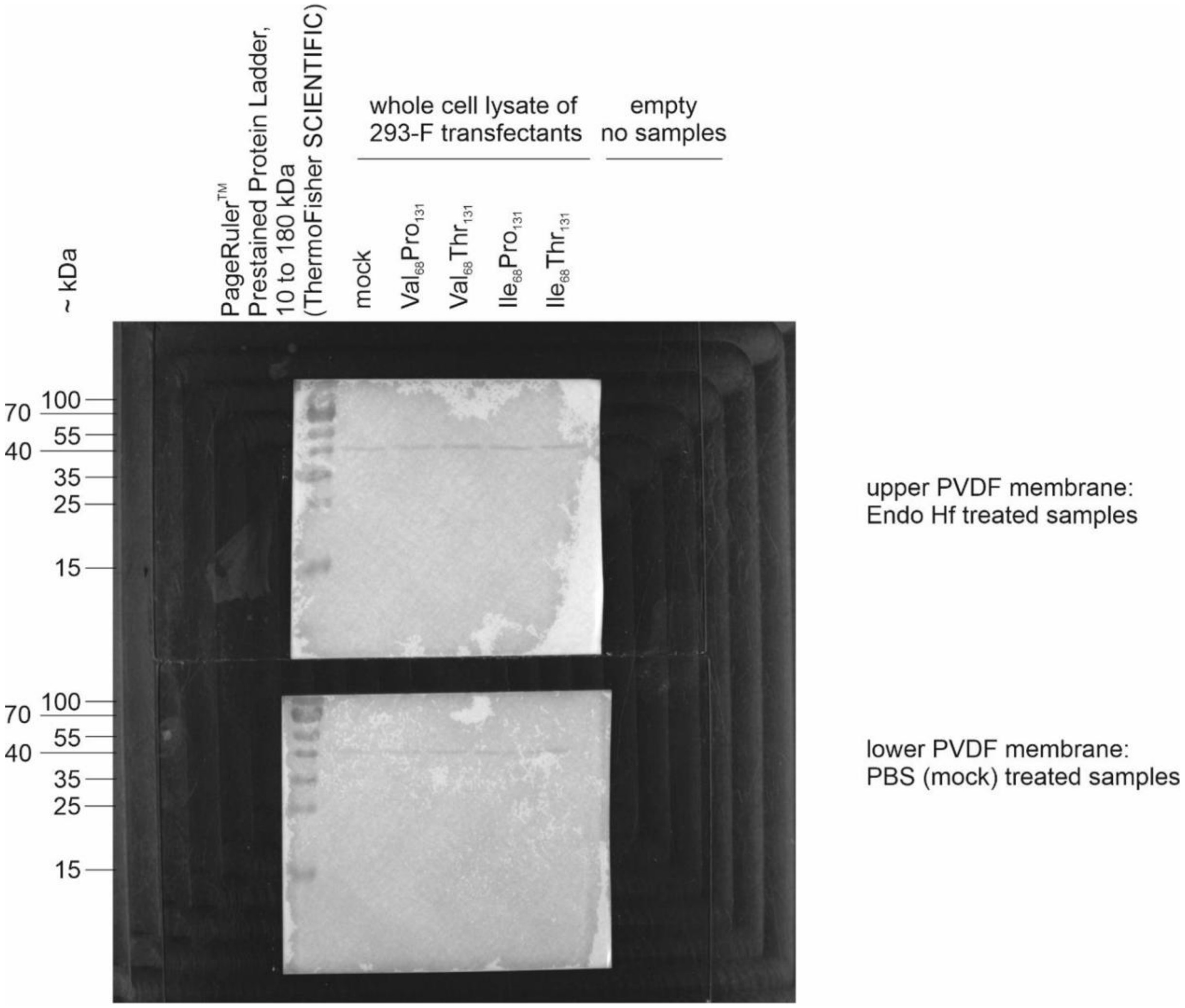

Picture was taken using weak white light in a FUSION SL apparatus (Vilber Lourmat) and the FusionCapt Advance SL4 16.09b software. Shutter was set to max (0.84) for two seconds.

Chemiluminescence

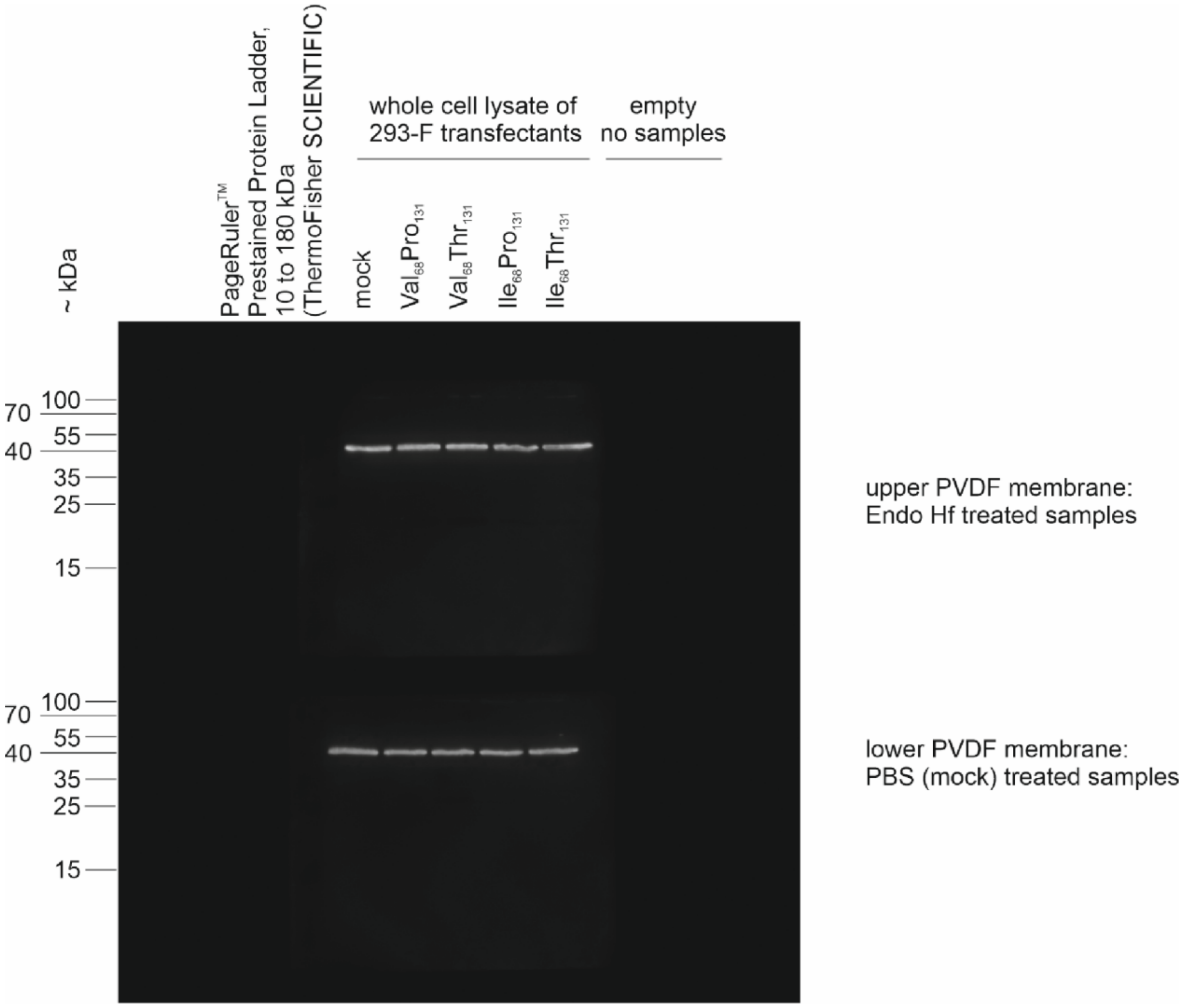

Picture was taken using the chemiluminescence function and the “full resolution” application for quantification in a FUSION SL apparatus (Vilber Lourmat) and the FusionCapt Advance SL4 16.09b software. Shutter was set to max (0.84) for two seconds test exposure. The auto exposure time was set to 34.4 seconds by the program.

Merge

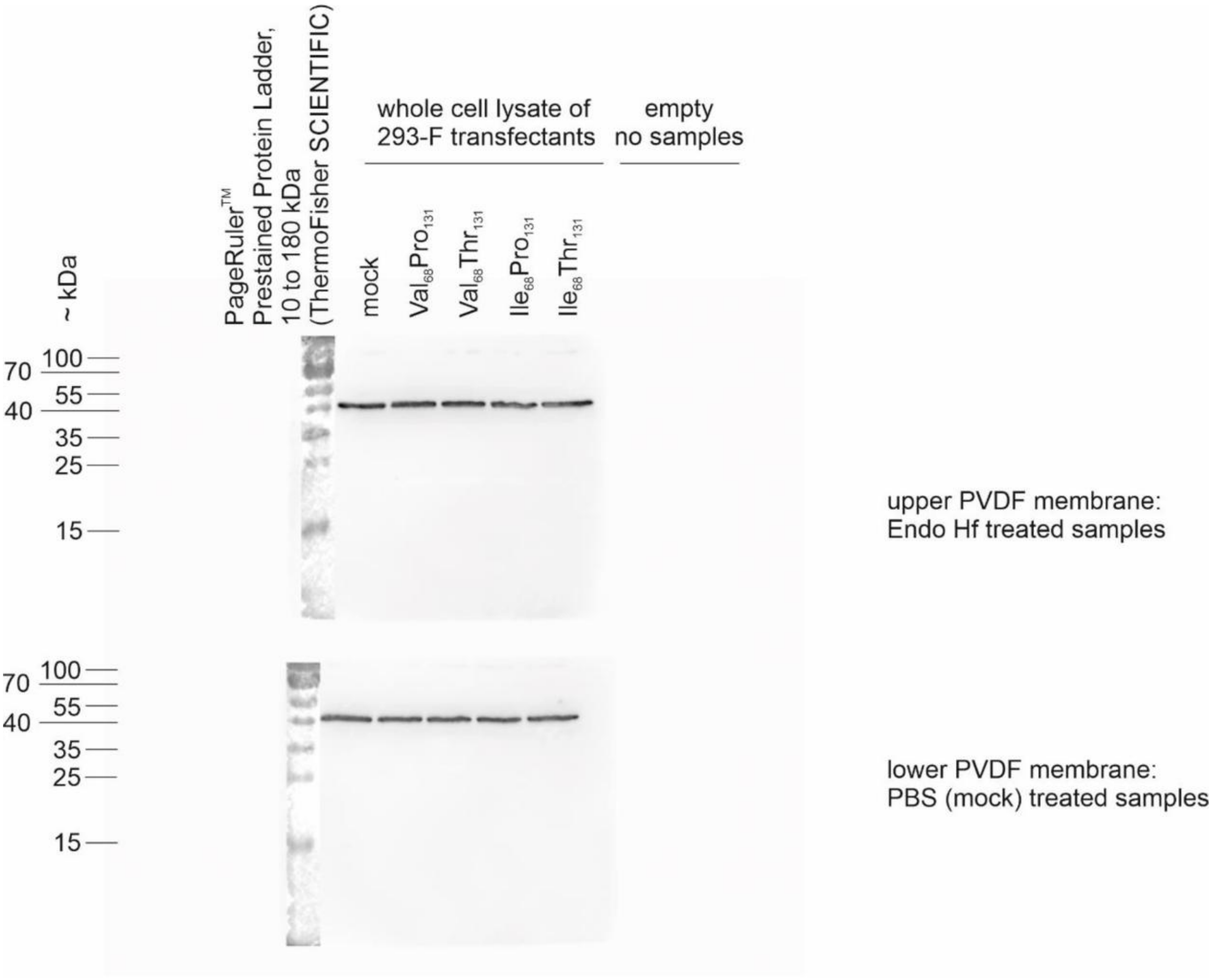

Using the FusionCapt Advance software, the chemiluminescence picture was inverted before the marker was merged using the implemented “multiple image processing” tool.
