# Supplementary material for: A common polymorphism in the human immunoreceptor NKp65 determines ligand interaction, cell surface expression and function
Source: PLoS One. 2025 Aug 13;20(8):e0329454. doi: 10.1371/journal.pone.0329454 (PMC12349009; doi:10.1371/journal.pone.0329454)
Supplement: S2 Table — The triplet XCT determines amino acid 131 of NKp65 that can be either threonine (X = Adenine) or proline (X = Cytosine). Adapted from Reference SNP (rs) Report of rs576601, National Library of Medicine (Status: 9/2022). Ref Allele = Reference Assembly allele; Alt Allele = Alternate allele; Ref HMOZ = Reference Homozygous genotype Frequency; Alt HMOZ = Alternate Homozygous genotype Frequency; HTRZ = Heterozygous genotype Frequency; HWEP = -Log(HWE Probability). (PDF) [file pone.0329454.s002.pdf]

Population	Group	Sample Size	Ref Allele	Alt Allele	Ref HMOZ	Alt HMOZ	HTRZ	HWEP
**Total**	Global	329510	C=0.32	A=0.68	0.11	0.46	0.43	32
**European**	Sub	286714	C=0.31	A=0.69	0.1	0.48	0.42	2
**African**	Sub	8704	C=0.60	A=0.40	0.36	0.16	0.48	0
**African Others**	Sub	318	C=0.69	A=0.31	0.48	0.11	0.41	0
**African American**	Sub	8386	C=0.60	A=0.40	0.36	0.16	0.48	0
**Asian**	Sub	6652	C=0.56	A=0.44	0.32	0.2	0.48	1
**East Asian**	Sub	4718	C=0.57	A=0.43	0.32	0.19	0.49	0
**Other Asian**	Sub	1934	C=0.54	A=0.46	0.30	0.21	0.49	0
**Latin American 1**	Sub	1042	C=0.36	A=0.64	0.14	0.43	0.43	1
**Latin American 2**	Sub	2988	C=0.32	A=0.68	0.10	0.46	0.44	0
**South Asian**	Sub	5200	C=0.30	A=0.70	0.10	0.49	0.41	1
**Other**	Sub	18210	C=0.36	A=0.64	0.14	0.41	0.45	3
