# Supplementary figures and images for: A common polymorphism in the human immunoreceptor NKp65 determines ligand interaction, cell surface expression and function
Source: PLoS One. 2025 Aug 13;20(8):e0329454. doi: 10.1371/journal.pone.0329454 (PMC12349009; doi:10.1371/journal.pone.0329454)

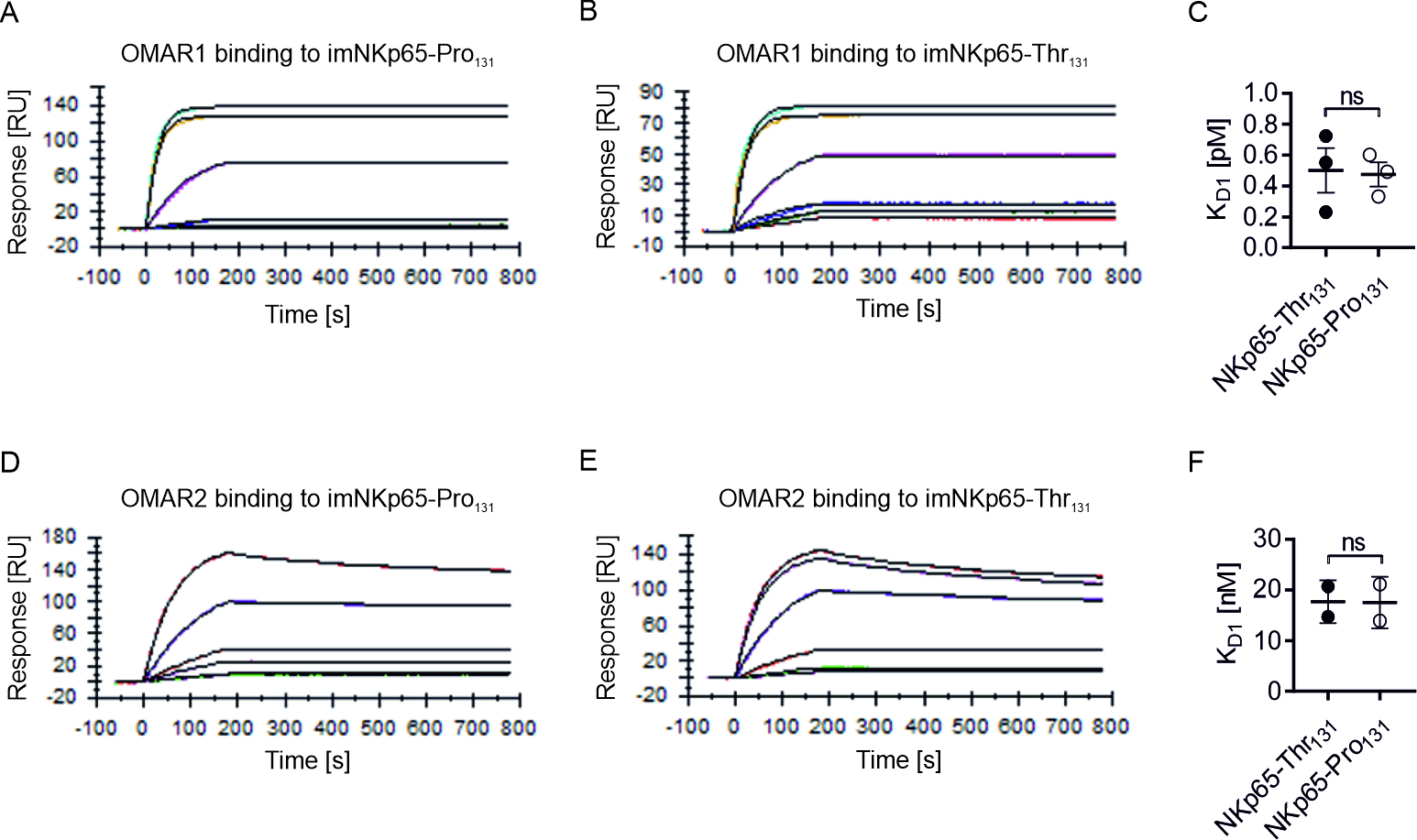

Supplement: S3 Fig — A and B, surface plasmon resonance spectroscopy multi-cycle kinetic measurement of mAb OMAR1 binding to immobilized NKp65. 49.6 ± 2.8 (A, NKp65-Pro131) and 48.1 ± 5.1 (B, NKp65-Thr131) RU of NKp65 were immobilized on a streptavidin chip and pulsed for 180 s with soluble mAb OMAR1 (1.25, 2.5, 5, 10 and 20 nM). Dissociation time was set to 600 s. Replicates (color) and bivalent fits (black) of one representative experiment are depicted. C, 45.3 ± 19.5 RU (Thr131) and 47.9 ± 20.6 RU (Pro131) of NKp65 were immobilized. Single values and mean ± SEM of KD1 of OMAR1 binding to NKp65 from two measurements are shown. A two tailed unpaired Student’s t-test was conducted, n = 3, p = 0.881 considered as not significant (ns); t = 0.1602, df = 4; F test to compare variances: F = 3.399, DFn = 2, Dfd = 2, p = 0.455. Recombinant protein was produced independently twice and measured three times on two different chip lots. D and E, surface plasmon resonance spectroscopy multi-cycle kinetic measurement of mAb OMAR2 binding to immobilized NKp65. 71.9 ± 1.8 (D, NKp65-Pro131) and 87.5 ± 5.3 (E, NKp65-Thr131) RU of NKp65 were immobilized on a streptavidin chip and pulsed for 180 s with soluble mAb OMAR2 (1.25, 2.5, 5, 10 and 20 nM). Dissociation time was set to 600 s. Replicates (color) and bivalent fits (black) of one representative experiment are depicted. F, 89.5 ± 5.4 RU (Thr131) and 52 ± 20.7 RU (Pro131) of NKp65 were immobilized. Single values and mean ± SEM of KD1 of OMAR2 binding to NKp65 are shown. A two tailed unpaired Student’s t-test was conducted, n = 2, p = 0,97 considered as not significant (ns); t = 0.04268, df = 2. Recombinant protein was produced independently twice and measured two times on two different chip lots. (TIF) [file pone.0329454.s003.tif]

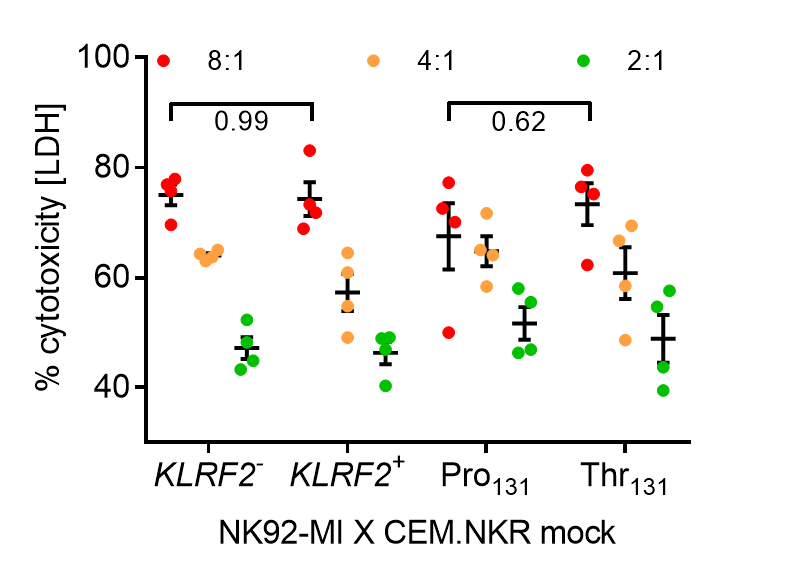

Supplement: S5 Fig — NKR-mock transductants. Cellular cytotoxicity of NK92-MI effector cells towards KACL-negative CEM.NKR-mock transductants after 4 h of co-culture at an E:T of 8:1, 4:1 and 2:1, measured by target cell LDH release. Depicted are single values and means ± SEM. Statistic was conducted using 2way ANOVA and Tukey’s multiple comparison test to analyze simple effects within columns (E:T ratio). Cell line (row factor) accounts for 0.74% of total variation, F (DFn, DFd) = 0.35 (3, 36), p = 0.79 and was considered not significant. E:T ratio (column factor) accounts for 69.52% of variation, F (DFn, DFd) = 49.52 (2, 36), p < 0.0001. No interaction of both factors was observed, F (DFn, DFd) = 1.060.585 (6, 36), p = 0.40. (TIF) [file pone.0329454.s005.tif]

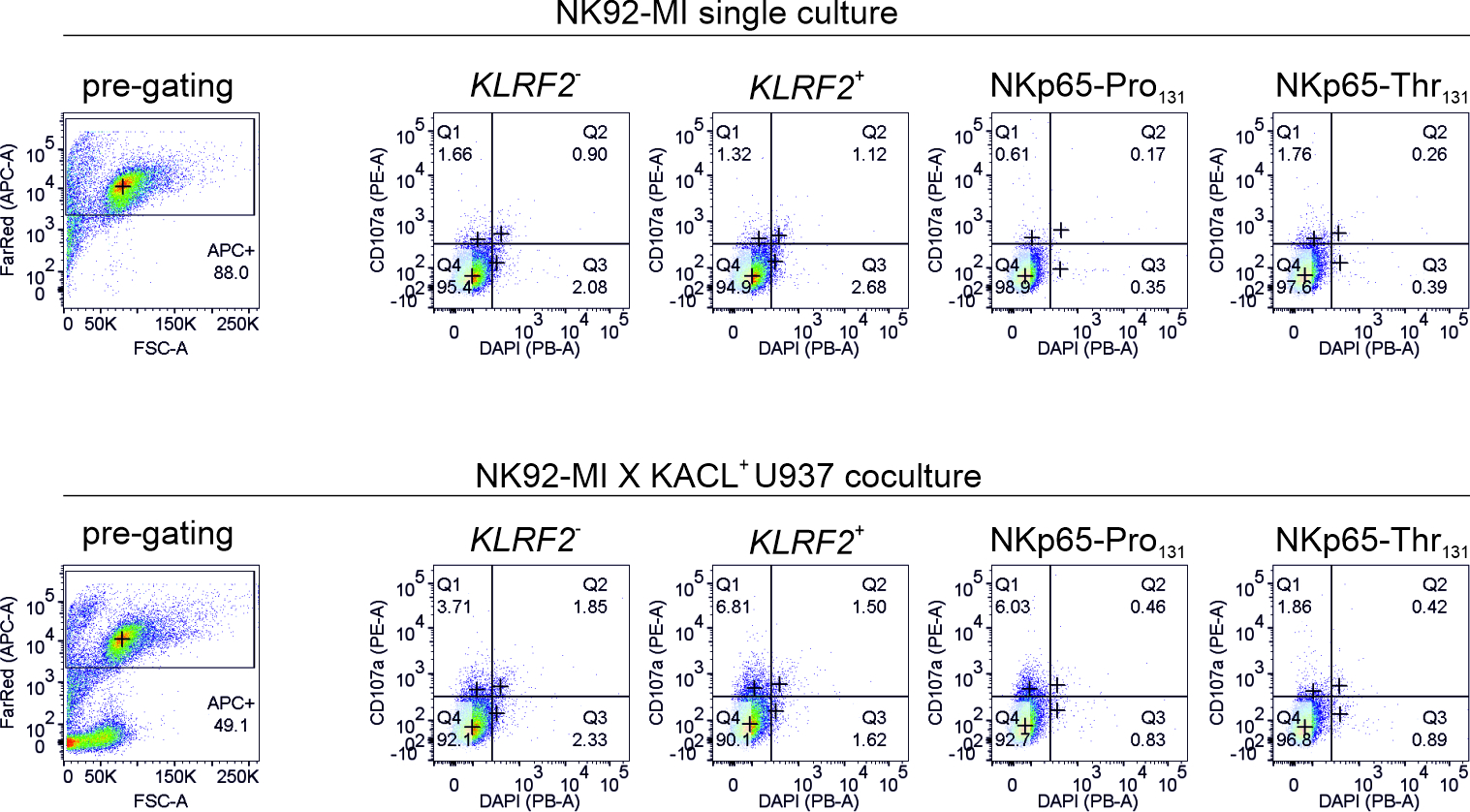

Supplement: S6 Fig — Effector cells were labeled with FarRed™ prior to single culture (top) or co-culture (bottom) with U937 cells. Subsequently, cells were stained with PE-conjugated anti CD107a antibody. Gate Q1 shows degranulation of viable NK92-MI effector cells. Shown are representative pseudocolor plots. Data are summarized in Fig 6A. (TIF) [file pone.0329454.s006.tif]

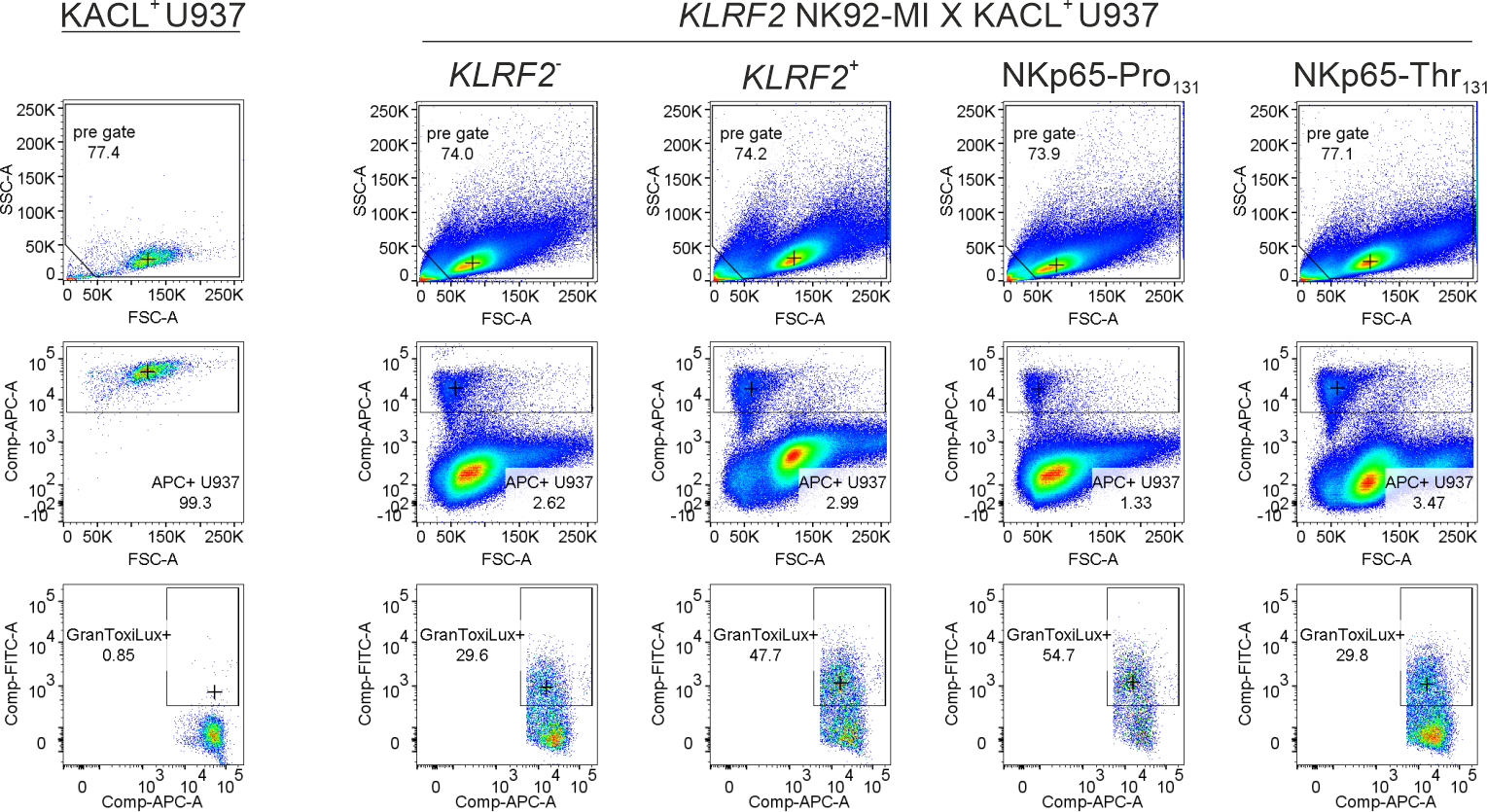

Supplement: S7 Fig — Target cells were labeled with TFL4 (APC) and cultured with (right) or without (left) NK92-MI effector cells in the presence of GranToxiLux® substrate for 4 h. Cleavage of GranToxiLux® by Granzyme B was measured in the FITC channel. Shown are representative pseudocolor plots from one of three independent experiments with technical triplicates. (TIF) [file pone.0329454.s007.tif]
