# Supplementary material for: A common polymorphism in the human immunoreceptor NKp65 determines ligand interaction, cell surface expression and function
Source: PLoS One. 2025 Aug 13;20(8):e0329454. doi: 10.1371/journal.pone.0329454 (PMC12349009; doi:10.1371/journal.pone.0329454)
Supplement: S4 Table — The triplet XTC determines the amino acid 68 of NKp65 that can be either valine (X = Guanine) or isoleucine (X = Adenine). Adapted from Reference SNP (rs) Report of rs1797517, National Library of Medicine (Status: 9/2022). Ref Allele = Reference Assembly allele; Alt Allele = Alternate allele; Ref HMOZ = Reference Homozygous genotype Frequency; Alt HMOZ = Alternate Homozygous genotype Frequency; HTRZ = Heterozygous genotype Frequency; HWEP = -Log(HWE Probability). (PDF) [file pone.0329454.s004.pdf]

Population	Group	Sample Size	Ref Allele	Alt Allele	Ref HMOZ	Alt HMOZ	HTRZ	HWEP
**Total**	Global	325116	G=0.24	A=0.76	0.06	0.59	0.35	14
**European**	Sub	280790	G=0.23	A=0.77	0.06	0.59	0.35	7
**African**	Sub	7040	G=0.35	A=0.65	0.13	0.44	0.43	4
**African Others**	Sub	250	G=0.38	A=0.62	0.14	0.38	0.48	0
**African American**	Sub	6790	G=0.35	A=0.65	0.13	0.44	0.43	4
**Asian**	Sub	6812	G=0.28	A=0.72	0.08	0.52	0.40	0
**East Asian**	Sub	4870	G=0.29	A=0.71	0.08	0.50	0.42	0
**Other Asian**	Sub	1942	G=0.25	A=0.75	0.06	0.57	0.37	0
**Latin American 1**	Sub	994	G=0.25	A=0.75	0.07	0.57	0.36	1
**Latin American 2**	Sub	6646	G=0.24	A=0.76	0.06	0.58	0.36	2
**South Asian**	Sub	5132	G=0.20	A=0.80	0.05	0.65	0.30	2
**Other**	Sub	17702	G=0.24	A=0.76	0.06	0.58	0.36	0
